# A Technique of Forced Expiratory Noise Time Evaluation Provides Distinguishing Human Pulmonary Ventilation Dynamics During Long-Term Head-Down and Head-Up Tilt Bed Rest Tests Simulating Micro and Lunar Gravity

**DOI:** 10.3389/fphys.2018.01255

**Published:** 2018-10-01

**Authors:** Veronika V. Malaeva, Vladimir I. Korenbaum, Irina A. Pochekutova, Anatoly E. Kostiv, Svetlana N. Shin, Vladimir P. Katuntsev, Viktor M. Baranov

**Affiliations:** ^1^Department of Acoustic Tomography, V. I. Il’ichev Pacific Oceanological Institute, Far Eastern Branch of Russian Academy of Sciences, Vladivostok, Russia; ^2^Research Institute for Space Medicine, Federal Biomedical Agency of Russia, Federal Research Clinical Center, Moscow, Russia

**Keywords:** lung ventilation, forced exhalation, noise duration, signal processing, head inclination, weightlessness, simulation

## Abstract

Estimating the effect of microgravity/hypogravity on pulmonary ventilation function remains topical. Recently developed acoustic techniques based on the evaluation of the forced expiratory noise time (FETa) were hypothesized to be a promising tool for this aim. The aim of the protocol is to study the effect of two different modalities of bed rest space simulations (microgravity and lunar gravity) on FETa and spirometric indices. The FETa in the frequency band of 200–2000 Hz, recorded above human trachea, was evaluated. The 21st-day exposure to 6 degree head-down tilt (HDT) bed rest, simulating microgravity, and 9.6 degree head-up tilt (HUT) bed rest with head-zero tilt (HZT) rest intervals (HUT + HZT), simulating lunar gravity, in statistically identical subgroups of five and six healthy male volunteers, was studied. In the course of HDT bed rest, a significant elongation of FETa was found in relation to background measurements in “sitting” position (*p* = 0.016). The effect corresponded to a significant decrease of basic spirometric indices (*p* < 0.02). Moreover, FETa provided reliable discrimination of HDT and HUT + HZT bed rest tests (*p* = 0.018), while spirometric indices did not (*p* > 0.05). Based on previously found correlations (Korenbaum and Pochekutova, 2008; Malaeva et al., 2017), a FETa elongation in response to HDT bed rest was attributed to an increase of aerodynamic resistance of the respiratory tract. The technique seems promising to monitor human pulmonary ventilation dynamics in long-term space missions; however, new studies are welcome to verify it in real spaceflight.

## Introduction

It is well known that in microgravity, a redistribution of body fluids to the cranial direction takes place. Blood moves from the lower extremities to the abdominal cavity and thorax (Gazenko et al., 1997; Prisk, 2000) increasing a blood filling of lungs (Grigor’ev and Egorov, 1988; West et al., 1997). The diaphragm forms a more convex shape limiting lung volumes. These structural and functional changes caused by gravity discharge may result in alterations of pulmonary ventilation and gas exchange (West et al., 1997; Prisk, 2000; Prisk et al., 2002; Baranov, 2011; Watenpaugh, 2016).

There were signs of respiratory discomfort and respiratory diseases among Russian cosmonauts and American astronauts in prolonged spaceflights (Gazenko and Grigor’ev, 1980; West et al., 1997; Goncharov et al., 2001). Although Prisk (2014) reported on the increasing number of astronauts performing long-term low-orbital missions on the International Space Station up to 6 months and even longer without showing severe respiratory problems, this kind of disorder could not be excluded in the long-term autonomous flights to the outer space. Thus, the problem of estimating the effect of microgravity/hypogravity on pulmonary ventilation remains topical.

The recently developed acoustic technique (Korenbaum et al., 2013) based on the objective evaluation of forced expiratory noise time (FETa) recorded at the neck region above the human trachea seems to be a promising tool for this aim. Diagnostic sensitivity and specificity of FETa technique were previously estimated to be near 90% in the sample, consisting of young male asthma patients with bronchial obstruction confirmed by spirometry, and the referent group of young healthy males (Korenbaum et al., 2012). The technique has shown an ability of identifying hidden (spirometry negative) bronchial obstruction (Pochekutova and Korenbaum, 2013) and certain potential in monitoring pulmonary ventilation function in subjects exposed to extreme environmental factors (Pochekutova and Korenbaum, 2011).

It is hypothesized that changing the configuration of the chest, lung volumes, and biomechanical properties of the lungs and respiratory tract during prolonged space missions should lead to a change in acoustic characteristics of forced exhalation (FE). However, the hypothesis has to be preliminary tested in ground-based conditions.

The bed rest tests are widely used to simulate the physiological effects of microgravity (Prisk et al., 2002; Morukov and Vasil’ev, 2013). In particular, 6 degree head-down tilt (HDT) bed rest (Prisk et al., 2002; Morukov and Vasil’ev, 2013; Watenpaugh, 2016) is used to simulate the microgravity. Recently, a method of 9.6° head-up tilt (HUT) bed rest has been developed to simulate physiologic effects of lunar gravity (Baranov et al., 2016).

The aim of the protocol is to study the effect of two different modalities of long-term bed rest space simulations (6 degree HDT bed rest simulating microgravity, and 9.6 degree HUT simulating lunar gravity) on FETa and spirometric indices. It should be mentioned that the current protocol was a part of a multidisciplinary «Selena» Bed Rest Study, some results of which were already published (Cherepov et al., 2014; Segizbaeva et al., 2016).

## Materials and Methods

### Ethics Statement

The experimental part of the study was performed in the Federal Research Clinical Center for Specialized Types of Medical Care and Medical Technologies of the Federal Biomedical Agency of Russia. The scientific research program was approved by the Ethics Committee permission No. 4 of February 5, 2015.

Volunteers were acquainted with procedure demands and signed informed consents to participate in the study as tested subjects. The informed consent provided for a refusal of any subject to continue participating in the trial, by his request.

### Sample

Eleven healthy male volunteers participated in the study. Only one of them was a smoker with the smoking index of three packs per year, but he did not smoke during the experiment. Each volunteer involvement in the experiment was admitted by the medical expert commission.

Age of volunteers was (Me; UQ; LQ) 24; 19; 32 years, height–175; 173; 181 cm, and body mass–72; 63; 73 kg. The volunteers were arbitrarily divided into two subgroups (five and six subjects), which did not differ significantly by age, anthropometric data, and spirometric indices of lung ventilation function (**Table 1**).

**Table 1:** Age and anthropometric data in the HDT and HUT + HZT test subgroups 2 days before starting bed rest.

Parameters, Me; LQ; UQ	HDT subgroup, *n* = 5	HUT + HZT subgroup, *n* = 6	Mann–Whitney U test (*p*)
Age, yr	22; 21; 24	25; 19; 32	0.644
Height, cm	173; 172; 176	176; 173; 181	0.356
Weight, kg	70; 62; 73	72; 66; 73	0.518
VC, l	5.46; 5.23; 6.26	5.38; 5.23; 5.85	0.791
FEV1, l	4.67; 4.28; 5.22	4.49; 4.25; 5.10	0.584
FEV1/FVC, %	87; 77; 91	80; 77; 88	0.599
MMEF, l/s	3.79; 3.56; 6.38	4.05; 3.66; 5.48	0.309
FETa, s	1.17; 1.05; 1.94	1.50; 1.13; 2.26	0.064

The first subgroup, consisting of five subjects, participated in the 6 degree HDT bed rest simulating physiologic effects of microgravity during 21 days (exp_1). The second subgroup (exp_2), consisting of six subjects, was also in the 6 degree HDT position during the first 5 days to simulate manned flight to the moon in microgravity. However, on the sixth day, they were transferred to bed rest with a positive head inclination of +9.6 degree with respect to the horizon (HUT), imitating the physiological effects of lunar gravity. Subjects of this subgroup were in HUT position for the next 16 days, daily from 7 am to 11 pm. However, for the night sleep (from 11 pm to 7 am), they were moved to the horizontal position with head-zero tilt (HZT) to simulate night rest of the lunar expedition. Thus, the second subgroup was in 9.6 degree HDT + HZT bed rest during the last 16 days of the exp_2.

Subjects were fed four times per day while being 10–15 min in the horizontal elbow position with a torso turning at the angle of 45° to the tray with food. The subjects of both groups took a vertical position every day for 15 min before bedtime to fulfill hygiene procedures. Thus, a total deviation from the body positions under study (HDT or HUT + HZT) for each subject was 55–75 min per day (i.e., no more than 5.2% of total experiment duration). Special monitoring of subjects lying in studied positions was carried out by the round-the-clock video surveillance and instant exams of physicians.

A study of the pulmonary ventilation function was preceded by the careful training of all subjects to fulfill the slow volume capacity (VC) and FE maneuvers correctly. After arrival at the clinics, initial measurements for all subjects were made in the “sitting” position 2 days before starting bed rest. During bed rest, measurements were performed in the HDT and/or HUT positions of subjects on the 3rd, 6th, 9th, 14th, and 20th days of each experiment in the same daytime interval. The final measurements for all subjects were made in the “sitting” position 2 days after ending bed rest.

### Measures

Forced expiratory noises were picked up by the acoustic sensor containing electret microphone (W62A, Panasonic) provided with a stethoscope conical head with an opening angle of 120°, 20 mm at its base diameter, and 5 mm in depth (Korenbaum et al., 2008). Specially designed software PPhT-3.1.12 (V. I. Il’ichev Pacific Oceanological Institute, Far Eastern Branch, Russian Academy of Sciences) was used to introduce noise signals through the microphone input of the 16-bit outer sound card (Transit, M-Audio) of a portable computer with sampling frequency 8 kHz and to measure the FETa (Korenbaum et al., 2013). Evaluation of the FETa for each recorded file was performed by using specially developed algorithm.

**FIGURE 1:**
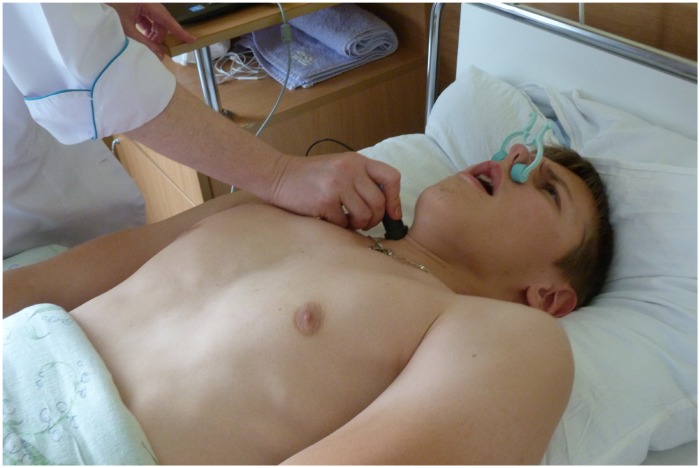
The procedure of tracheal forced expiratory noise recording in bed rest (a written informed consent for the publication of this image was obtained from the volunteer).

The acoustic sensor was placed on subject’s right larynx area, inwardly from the anterior edge of their sternocleidomastoid muscle; a clamp was applied on the nose. The sensor was applied close to the soft tissues by the stethoscopic head, and the operator making noise recording held the box with her hand (**Figure 1**). Each subject performed a forced expiratory maneuver from a position of maximal inspiration. He held breathing between inspiration and expiration for 0.5–1 s. In order to carry out the maneuver properly, a maximum sharp and maximum complete expiration were required. The experienced pulmonary function physician (VM) monitored the forced expiratory performance. Three to five well-done attempts were saved (**Supplementary Table S1**). The best attempt was chosen by the maximum FETa value.

A spirometry was performed by a standard procedure. Spirometric indices VC, forced VC (FVC), forced expiratory volume for 1 s (FEV1), FEV1/FVC ratio, and maximal midexpiratory flow (MMEF) were assessed for each subject. Spirometry was carried out with spirometer MicroLoop (MicroMedical Ltd., Rochester, United Kingdom). The best attempts were selected by the greatest sum of FEV1 + FVC. Current ATS/ERS criteria for acceptability were used. Three to six repeatable attempts were saved (**Supplementary Table S2**).

It is noteworthy that tracheal noises and spirometry were recorded in different attempts of FE (while in the same position of each subject) as the interaction of expiratory airflow with flow meter armature may result in the occurrence of adventitious noises changing FETa value determined above trachea (Malaeva et al., 2015).

### Data Analysis and Statistics

An evaluation of the FETa in each recorded acoustic file was performed by the developed algorithm (Korenbaum et al., 2012). According to it, filtration was carried out in the frequency band of 200–2000 Hz with a Kaiser Windowed Direct-form Finite Impulse Response filter; the FE waveform envelope was constructed doubly in the forward and opposite directions by the moving average method with an accumulation period of 0.01 s. Then, peak amplitude (A) of the envelope was calculated. At the threshold level *L* = 0.005 A, the times of beginning T1 and ending T2 of the FE noise process were measured by envelope when moving from the peak to the left and to the right. Time T1 was fixed by the program quite reliably. When the ending time (T2) was estimated, the first of feasible roots was determined by the program although a skilled operator sometimes may tell that noise process was still in progress. To eliminate this effect, a semiautomated T2 evaluation procedure was used in which the program calculated consequently all roots of the equation *L* = 0.005 A automatically. However, the operator, displacing the cursor interactively along the calculated roots on the plot next by next, had a chance of selecting the root that corresponded to the ending of noise process subjectively. Meanwhile, it should be noted that 100% of the estimates used for analysis of the sample engaged the first T2 root, which was evaluated automatically without any manual adjustment and collected. Since T1, T2 had been measured automatically or semiautomatically, the program automatically calculated the duration of tracheal FE noises as the difference FETa = T2-T1.

A variability of FETa estimates in repeated FE maneuvers made by the same well-trained healthy subjects was characterized by the coefficient of variation (SD/mean) typically being below 10% (Pochekutova and Korenbaum, 2014).

A group dynamics of FETa and spirometric indices was statistically assessed with Statistica (StatSoft Inc.,). The specific procedure of statistical data analysis is implemented. The first consideration in the basis of the procedure is that nonparametric methods are preferable for small sample analysis because assessment of normality or non-normality of distribution is very unreliable here. Therefore, nonparametric Mann–Whitney U test was used when comparing independent subgroups and nonparametric Wilcoxon T test was applied when comparing the best attempts for dependent samples. However, the smallness of the samples (*n*) does not allow a sufficiently fine analysis of the experiments with repeated attempts. This possibility is provided only by the two-factor ANOVA (for example, Factorial ANOVA). Unfortunately, the latter does not have its nonparametric analog. Therefore, it is necessary to use the estimates used in the Factorial ANOVA module of Statistica software where there are no other parameters except means and 95% confidence interval (CI). It is well known that ANOVA is more limited not by the requirement of normality of distributions, but by the conditions of variance homogeneity. That is why Factorial ANOVA is planned to be used, testing assumptions on variance homogeneity. Additionally nonparametric Mann–Whitney U and Wald–Wolfowitz serial tests for independent samples are planned to use to recheck results of Factorial ANOVA when assumptions on variance homogeneity are not met.

The threshold of statistical significance was established at *p* < 0.05.

To prevent from biasing, subjects were arbitrarily divided between the two subgroups. It was not known *a priori* whether there will be a difference between HDT and HUT + HZT bed rest tests and in which direction. Therefore, when measuring, neither the expectations of the subjects nor the investigator (VM) should contribute to the bias of the results in recording acoustic signals (wave files). Processing of recorded acoustic signals was made by an independent investigator (SS) who did not know in which subgroup was included the subject whose files were being processed. Thus, the study was blinded in this part. Furthermore, the FETa estimates were made automatically by means of the algorithm described earlier. Thus, an influence of the investigators and subjects on FETa estimates was excluded.

## Results

### Sample Characterization

There were no significant differences in age, anthropometric data, as well as in acoustic and spirometric parameters between subgroups involved in the HDT and the HUT + HZT bed rest tests 2 days before starting hypokinesia (**Table 1**). Similarly, there were no differences in acoustic and spirometric parameters between the HDT and HUT + HZT subgroups 2 days after finishing bed rest (data are not shown).

Individual intrasession variability of the measured FETa values was assessed in the whole group as (Me, LQ, UQ) 5.94%, 3.02%, and 8.37% (**Supplementary Table S1**).

As for the best attempts, a difference of parameters was analyzed between their meanings at 2 days before starting and at 2 days after finishing bed rest, when all 11 subjects were tested in “sitting” position. There were no significant differences in FETa as well as in spirometric indices according to Wilcoxon T test (**Table 2**).

**Table 2:** Values of FETa and spirometric indices in the whole group of subjects (*n* = 11) before and after bed rest.

Parameters, Me; LQ; UQ	Before	After	Wilcoxon T test p
VC, l	5.44; 5.29; 6.30	5.68; 5.25; 6.28	0.050
FEV1, l	4.68; 4.36; 5.16	4.97; 4.35; 5.16	0.050
FEV1/FVC, %	82; 78; 92	84; 79; 89	0.131
MMEF, l/s	3.84; 3.58; 5.96	4.12; 3.88; 5.86	0.062
FETa, s	1.40; 1.12; 2.03	1.47; 1.13; 1.94	0.25

### HDT Impact Estimation in the Whole Group

Since on the third day of the experiment, all 11 subjects of both subgroups were in the HDT position, whole group comparisons of FETa and spirometric indices for the best attempts were performed in relation to the background (2 days before starting hypokinesia), when the same persons were in the “sitting” position (**Table 3**). A significant increase was found in FETa. On the contrary, spirometric indices decreased essentially. However, individual spirometric indices did not reach the limits of individual predicted values.

**Table 3:** Values of FETa and spirometric indices in the whole group of subjects (*n* = 11) on the third day of the HDT bed rest and at 2 days before starting bed rest.

Parameters, Me; LQ; UQ	Before	3rd day	Wilcoxon T test; p
FETa, s	1.50; 1.13; 2.05	1.86; 1.32; 2.32	0.016
VC, l	5.44; 5.29; 6.30	5.31; 5.08; 6.11	0.020
FEV1, l	4.68; 4.36; 5.16	4.63; 4.16; 5.01	0.005
FEV1/FVC, %	82; 78; 92	78; 77; 89	0.003
MMEF, l/s	3.84; 3.58; 5.96	3.48; 3.30; 5.69	0.005

### Differences Between Subgroups

Two-factor ANOVA, as mentioned earlier, is a more informative method to assess the difference in repeated measurements of FETa and spirometric indices for two modalities–microgravity simulated by the 6 degree HDT bed rest (exp_1) vs. lunar gravity simulated by the 9.6 degree HUT + HZT bed rest (exp_2).

For acoustic parameter FETa, results of Factorial ANOVA (Statistica, StatSoft Inc.) with two factors–“exp_type” (exp_1 or exp_2) and “Day” of study–showed, that there is a statistically significant difference of FETa by the factor “exp_type” according to F test (*p* = 0.016). Data are not shown. Thus, an integral difference of FETa between two types of experiments–exp_1 (the HDT bed rest) and exp_2 (the HUT + HZT bed rest)–is revealed. On the contrary, there are no significant distinctions of FETa by the factor “Day” and by the combination of two factors “exp_type^∗^Day”.

**FIGURE 2:**
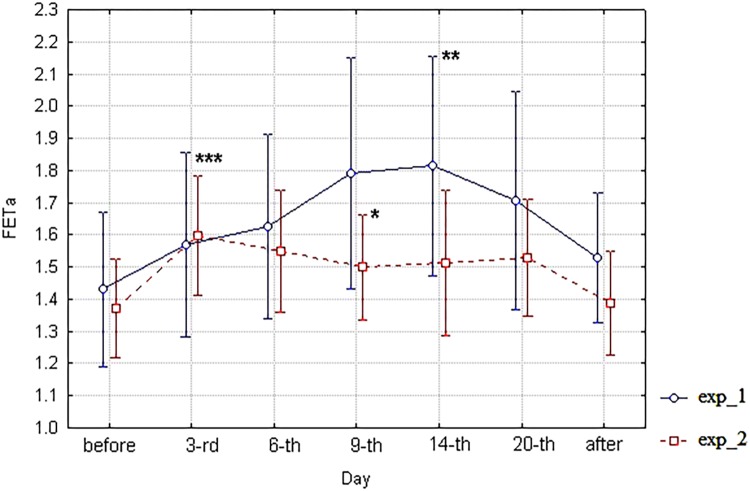
The diagram of FETa (unweighted means with 95% CI) by days. For exp_1–circles; for exp_2–squares; and ^∗^significant differences between exp_1 and exp_2 on the ninth day; and ^∗∗^ significant differences of FETa between the 3-rd and the 14-th days of exp_1; and ^∗∗∗^ significant differences of FETa between the 3-rd day of experiment and background (before) in the whole group (*n* = 11).

The “Day” differences of FETa between exp_1 and exp_2 after splitting the common procedure into 6 degree HDT and 9.6 degree HUT + HZT tests (from the sixth day of trial) are evidently seen in the diagram (**Figure 2**), where unweighted means of FETa are shown with limits of 95% CI for each subgroup (HDT vs. HUT + HZT) and consequently both types of experiments, and all days of study. The difference is especially amplified by the 9th–14th days of the trial. Furthermore, a FETa branch for the HDT bed rest (exp_1) is higher than a FETa branch for the HUT + HZT bed rest (exp_2).

The statistical significance of the day differences in the frame of ANOVA may be assessed with Fisher’s least significant difference test. The most interesting result of the test is the statistically significant difference in FETa between exp_1 and exp_2 subgroups on the 9th and 14th days (*p* = 0.041). However, there is no significant difference between subgroups before starting bed rest and after its finishing. Data are not shown. Unfortunately, the FETa parameter does not meet the ANOVA basic assumptions on homogeneity of variance (tests of homogeneity of variances *p* = 0.013, Levene’s test *p* = 0.000). Therefore, the analysis of significance of the day differences between exp_1 and exp_2 carried out within the framework of ANOVA should be considered as a preliminary one and demands further verification.

In order to assess the statistical significance of the day differences between exp_1 and exp_2, an additional procedure of nonparametric analysis (not demanding any assumptions on normality and homogeneity of samples) was applied involving the whole set of individual attempts of respiratory maneuvers. This approach provides for enlarging the volume of analyzed samples in relation to the best attempts analysis (**Tables 2**, **3**). However, due to the varying number of individual attempts, the approach demands using nonparametric tests for independent variants. Initially, the Mann–Whitney U test was used, and if there was no significant difference in it, the Wald–Wolfowitz serial test was applied.

This approach provided revealing statistically significant differences in FETa values between exp_1 and exp_2 on the ninth day of study (Wald–Wolfowitz serial test *p* = 0.018), which is marked by ^∗^ in **Figure 2**. Furthermore a statistically significant difference of FETa between the 3-rd and the 14-th days of exp_1 was revealed (Wald–Wolfowitz serial test *p* = 0.0026) which is marked ^∗∗^ in **Figure 2**. Also a statistically significant difference of FETa between the 3-rd day of experiment and background (before) in the whole group (*n* = 11) was found (Mann–Whitney U test *p* = 0.0163) which is marked ^∗∗∗^ in **Figure 2**. It is important that the FETa value for exp_1 is higher than in exp_2.

Similarly, two-factor ANOVA was applied to spirometric indices VC, FEV1, FEV1/FVC, and MMEF. For VC index, Factorial ANOVA with two factors–“exp_type” (exp_1 or exp_2) and “Day” of study–did not reveal any significant difference, though ANOVA basic assumptions were partially implemented here (tests of homogeneity of variances *p* = 0.007, Levene’s test *p* = 0.052). For FEV1 index, Factorial ANOVA with two factors–“exp_type” (exp_1 or exp_2) and “Day” of study–did not reveal any significant differences, though both ANOVA basic assumptions were implemented here (tests of homogeneity of variances *p* = 0.52, Levene’s test *p* = 0.08). Similar situation is observed for the FEV1/FVC index. For the MMEF index, Factorial ANOVA with two factors–“exp_type” (exp_1 or exp_2) and “Day” of study–did not reveal any significant differences, though ANOVA basic assumptions were partially implemented here (tests of homogeneity of variances *p* = 0.94, Levene’s test *p* = 0.009). Data of spirometric indices analysis are not shown.

### Clinical Observations

It should be noted that there were found no significant differences for any spirometric index between exp_1 and exp_2 subgroups 2 days before starting hypokinesia, on any day of bed rest simulation, and 2 days after finishing it. However, according to clinical observations, there was a different occurrence of respiratory complaints in the HDT subgroup (exp_1) and in the HUT + HZT subgroup (exp_2). In the HDT subgroup, respiratory complaints were registered in all participants of the experiment (5/5). They involved upper respiratory tract (nasal congestion)–5/5 and upper and lower lying respiratory tract (cough and nasal congestion)–1/5. On the contrary, in the HUT + HZT subgroup (exp_2), respiratory complaints were found only in two participants (2/6). They involved upper respiratory tract–1/6 and upper and lower lying respiratory tract–1/6. Furthermore, there were different trends of total respiratory complaints count in the subgroups during experiment (**Table 4**).

**Table 4:** Respiratory complaints count in exp_1 and exp_2 subgroups by days.

exp_type	Before	3rd day	6th day	9th day	14th day	20th day	After
exp_1 (*n* = 5)	0/5	3/5	4/5	5/5	4/5	3/5	2/5
exp_2 (*n* = 6)	0/6	2/6	0/6	1/6	1/6	1/6	0/6

Both subgroups replied to initial HDT bed rest (third day) by an appearance of respiratory complaints. There was additional increase in respiratory complaints count by the ninth day of HDT bed rest in the exp_1 subgroup, which was followed by its relative decrease to the end of this trial. In contrast, there was no subsequent change in the count of respiratory complaints for the exp_2 subgroup, despite its relative decrease after moving from HDT to HUT + HZT bed rest (sixth day).

## Discussion

The 6 degree HDT microgravity simulation is widely extended (Prisk, 2000; Morukov and Vasil’ev, 2013; Watenpaugh, 2016). However, Prisk et al. (2002) questioned a possibility of using the 6 degree HDT test as a microgravity model for the pulmonary function, since changes in the lung ventilation observed in real spaceflight did not agree with those detected in the HDT bed rest.

According to our study in the group of 11 healthy volunteers, a statistically significant decrease of spirometric indices VC, FEV1, FEV1/FVC, and MMEF is found on the third day of exposure to the 6 degree HDT bed rest in comparison with background values taken in the “sitting” position 2 days before starting the HDT. This is mostly in agreement with previous observations (Donina et al., 2013). Nevertheless, a significant increase of forced expiratory tracheal noise time, FETa, between these positions is revealed first time. It is noteworthy that 2 days after finishing the microgravity simulation, the mentioned indicators mostly returned to the background values obtained 2 days before starting simulation, confirming transient character of changes found.

Unfortunately, these observations do not answer the question whether the transient changes in FETa and spirometric indices are related to microgravity modeling or to pure postural effect of changing the posture from “sitting” to “lying” position. The key point to answer the question is to analyze subsequent dynamics of these indicators in two types of the experiment after its splitting into the HDT and the HUT + HZT branches, i.e., after transfer of the exp_2 subgroup from HDT to HUT + HZT position on the fifth day of the experiment.

**FIGURE 3:**
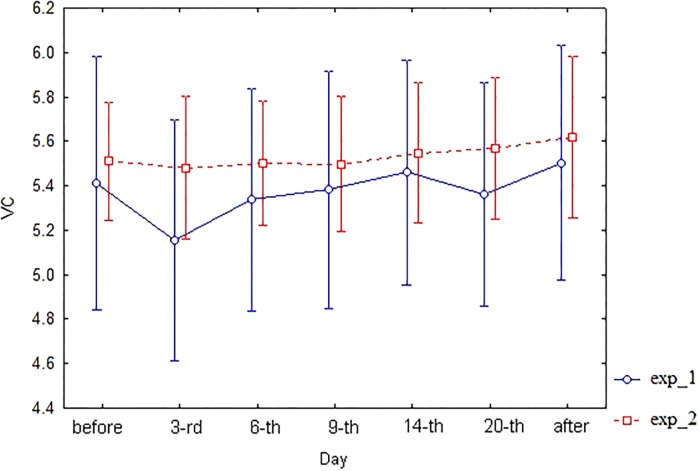
The diagram of VC (unweighted means with 95% CI) by days. For exp_1–circles; for exp_2–squares.

**FIGURE 4:**
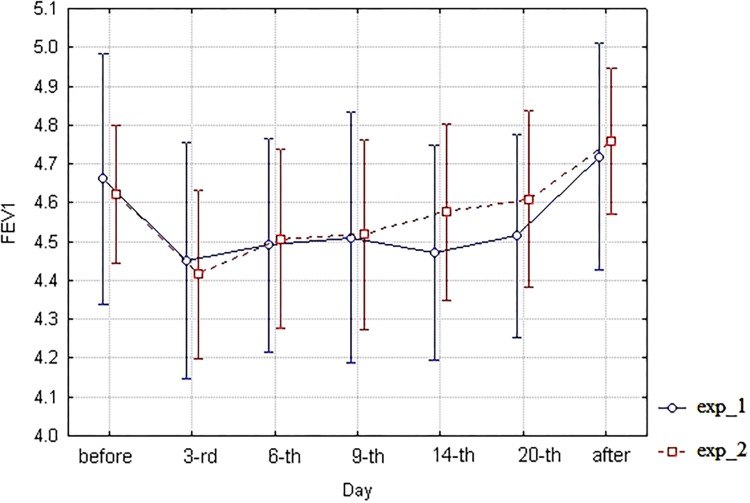
The diagram of FEV1 (unweighted means with 95% CI) by days. For exp_1–circles; for exp_2–squares.

**FIGURE 5:**
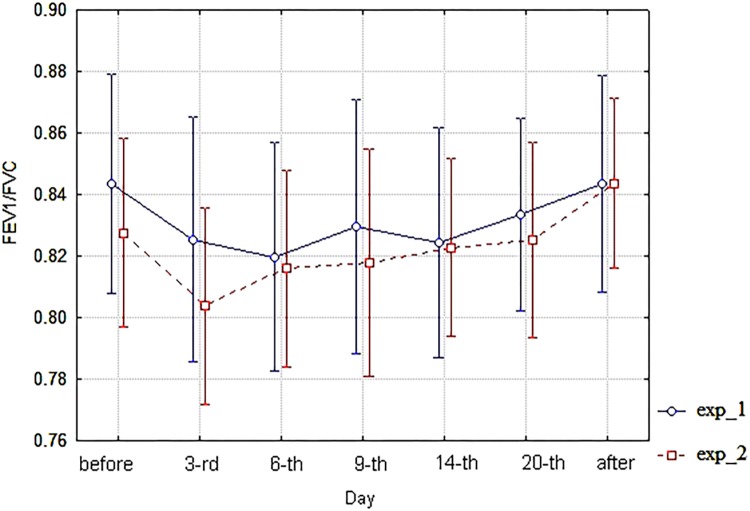
The diagram of FEV1/FVC (unweighted means with 95% CI) by days. For exp_1–circles; for exp_2–squares.

**FIGURE 6:**
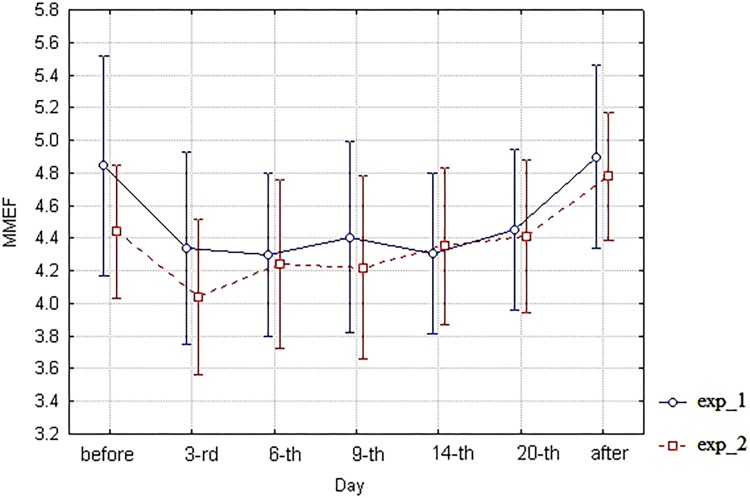
The diagram of MMEF (unweighted means with 95% CI) by days. For exp_1–circles; for exp_2–squares.

As for spirometric parameters, there were no statistically significant differences between the corresponding branches (exp_1 vs. exp_2) of diagrams of these indices within the 6th–20th day interval of bed rest simulation (**Figures 3**–**6**). It is in contradiction with observations (Meah and Tsigkopoulos, 2002), but significant differences between HUT and HDT positions were found in that study with enlarged head inclinations of more than ± 20 degrees.

Unlike spirometry, the acoustic parameter FETa (**Figure 2**) demonstrates an evident splitting of the curves for exp_1 and exp_2, reaching a statistical significance by the ninth day of simulation according to both two-factor ANOVA and Wald–Wolfowitz serial tests. It is noteworthy that the difference is achieved due to increase of FETa in the 6 degree HDT bed rest (exp_1) compared with the 9.6 degree HUT + HZT bed rest (exp_2).

It means that analyzed basic spirometric indices do not distinguish the 6 degree HDT and 9.6 degree HUT + HZT bed rest models, and, therefore, react primarily to the postural effect associated with changing “sitting” to “lying” posture. By the way, the result can partially explain the discrepancy between the responses of pulmonary ventilation indices in the 6 degree HDT test and in the microgravity condition of real spaceflight previously identified by Prisk et al. (2002).

On the contrary, FETa discriminates long-term influence of the 6 degree HDT and 9.6 degree HUT + HZT positions, and, therefore, allows one to distinguish the effect of 6 degree negative head end tilt from changing the “sitting” position to the “lying” one. It is the additional effect that actually simulates the microgravity.

The question is what kind of alterations in the pulmonary ventilation function is reflected by an increase of FETa under HDT bed rest?

It was previously demonstrated that FETa is proportional to aerodynamic resistance of airways. Initially, it was found theoretically (Korenbaum and Pochekutova, 2008). Later, it was experimentally verified by means of correlation with spirometry and body plethysmography-assessed parameters, including airway resistance (Korenbaum et al., 2016; Malaeva et al., 2017). Therefore, FETa increase as a response to the HDT bed rest may be mechanically treated as enlarging the aerodynamic resistance in the subjects of exp_1 subgroup. When breathing the same gas composition, an increase in aerodynamic resistance can be caused only by a decrease of lumen in the airways of respiratory tract. The decrease of airway lumen under FE is mechanically predicted in the range of bronchial tree levels between trachea and the seventh branching generation in accordance with estimations (Korenbaum et al., 2009).

Next, it should be noted that all subjects of exp_1 subgroup had some respiratory complaints, apparently caused by the influence of the HDT test. The most common were respiratory complaints that indicate swelling of the upper respiratory tract walls. Obviously, the swelling should lead to a decrease in airway lumen. Thus, we may mechanically speculate that the revealed dominant response of FETa elongation to the HDT test can be considered as a sign of the swelling phenomena in the walls, not only in upper, but also in the more distal airways. It is the effect that actually should be followed by an increase of aerodynamic resistance under FE in the mentioned levels of bronchial tree (Korenbaum et al., 2009).

Probably, an origin of the swelling may be associated with a redistribution of fluids in the human body in the course of the HDT bed rest (West et al., 1997; Donina et al., 2013). This speculation seems to be in qualitative agreement with the trend of increase of respiratory complaints count in the exp_1 subgroup between the third and the ninth days (**Table 4**).

On the contrary, in the exp_2 subgroup, respiratory complaints were observed only in 2/6 subjects. Probably, raising the head end in the course of the HUT + HZT bed rest resulted in a relative “outflow” of liquids compared with the HDT test (West et al., 1997; Donina et al., 2013). It should be followed by a reduction of swelling, and, consequently, a lower aerodynamic resistance of the respiratory tract in this subgroup, which, indeed, was reflected by a significant lower FETa value in comparison with the exp_1 subgroup as well as by lower respiratory complaints count in the exp_2 subgroup during HUT + HZT bed rest (**Table 4**).

Therefore, the basic spirometric indices do not distinguish 6 degree HDT bed rest, simulating microgravity, against the 9.6 degree HUT + HZT one, simulating lunar gravity. However, FETa successfully discriminates the microgravity and lunar gravity models and, in addition, allows one to monitor the change in aerodynamic resistance during the long-term 6 degree HDT bed rest. Thus, the acoustic method using FETa estimation makes it possible to evaluate the dynamics of pulmonary ventilation function in the course of ground-based simulation of microgravity by the 6 degree HDT and 9.6 degree HUT + HZT tests without any risk of contamination.

A limitation of the developed acoustic technique is the absence hitherto of the procedure of objective controlling of the correctness of the FE maneuver attempts, which resulted in increasing the FETa intra-individual variability compared with the basic spirometry indices (Pochekutova and Korenbaum, 2014). Therefore, a preliminary training of subjects under the guidance of an experienced operator is necessary. This is a disadvantage of the FETa acoustic technique for usage in medical diagnostics/screening. However, the disadvantage is partially parried by the repeated use of the technique in the sample, which has been already trained. It is seen in the low FETa intrasession variability found for most of the participants of current study (**Supplementary Table S1**). Thus, individual monitoring of pulmonary function including performed in extreme conditions such as space flights seems to be a pertinent application of the technique.

Nevertheless, additional studies are welcome to verify whether FETa is a more sensitive tool than basic spirometry to monitor individual dynamics of pulmonary ventilation function in real spaceflights.

## Conclusion

1. In the course of the 6 degree HDT bed rest, simulating microgravity, a significant elongation of the tracheal FETa in the frequency band of 200–2000 Hz was found on the third day of the experiment in the group of 11 subjects in relation to background measurement of the same subjects in “sitting” position made 2 days before starting the bed rest. The effect corresponded to a significant decrease in spirometric indices of lung ventilation function VC, FEV1, FEV1/FVC, and MMEF.
2. An evaluation of the tracheal FETa in the frequency band of 200–2000 Hz provided reliable discrimination of 6 degree HDT bed rest vs. 9.6 degree HUT bed rest (with zero head-tilt night rest intervals) being branches of the experiment, simulating long-term microgravity and lunar gravity in statistically identical subgroups, consisting of five and six subjects respectively, while the analyzed spirometric indices did not reveal significant distinctions between these tests (subgroups).
3. Since significant direct correlation of expiratory bronchial resistance with FETa in the frequency band of 200–2000 Hz was found previously (Korenbaum and Pochekutova, 2008; Malaeva et al., 2017), an elongation of the latter parameter in response to the 6 degree HDT bed rest simulating microgravity may be mechanically attributed to an increase of aerodynamic resistance of the respiratory tract that may occur due to decreasing airway lumen as a probable result of airway wall swelling.

## Author Contributions

VM collected all acoustic and spirometric data, participated in data processing and statistical analysis, formulated and discussed results, based conclusion No. 1, participated in the bases of conclusion No. 3, and drafted a part of the manuscript. VIK developed the method of FETa evaluation, suggested procedure and fulfilled statistical processing of data, made acoustical interpretation of results, based conclusions Nos. 2, 3, and drafted the manuscript. IP found and analyzed references, made physiological interpretation of results, participated in formulation and discussion of results, participated in conclusion No. 3 bases, and critically revised the manuscript for important intellectual content. AK developed experimental apparatus and software for FETa evaluation, used it for signal acquisition and processing, participated in developing design of experiment, and discussed basic results of the study. SS processed and analyzed acoustic data and participated in discussion of basic results of the study. VPK developed a design of long-term ground-based experiment with two types of hypokinesia and study of FETa possibilities to monitor subjects in models of microgravity and lunar gravity, participated in formulation of basic results of the study, and critically revised the manuscript for important intellectual content. VB hypothesized a possibility of applying FETa for the analysis of respiratory symptoms in cosmonauts and proposed conception for ground-based testing of this hypothesis, participated in formulation of basic results of the study, and critically revised the manuscript for important intellectual content. All authors approved the final version of the manuscript, agreed to be accountable for all aspects of the work in ensuring that questions related to the accuracy or integrity of any part of the work are appropriately investigated, and resolved that all persons designated as authors qualify for authorship, and all those who qualify for authorship are listed.

## Conflict of Interest Statement

The authors declare that the research was conducted in the absence of any commercial or financial relationships that could be construed as a potential conflict of interest.

## Supplementary Material

TABLE S1 Acoustic data EXP_type – exp_1 or exp_2, VOLUNTEER_cod – volunteer number, DAY of exp – day of experiment, DAY_cod – cod of the day of experiment, FETa – Forced expiratory noise time, Variability_session – intrasession variability (CV%) of FETa for every person in each day of experiment.

TABLE S2 Spirometry data EXP_type_SP – exp_1 or exp_2 for spirometry, VOLUNTEER_cod_SP – volunteer number for spirometry, DAY_exp_SP – day of experiment for spirometry, DAY_SP_cod – cod of the day of experiment for spirometry, FEV1 – FEV1, FVC – FVC, FEV1/FVC – FEV1/FVC MMEF – MMEF, VC – VC.
