# Supplementary material for: A Technique of Forced Expiratory Noise Time Evaluation Provides Distinguishing Human Pulmonary Ventilation Dynamics During Long-Term Head-Down and Head-Up Tilt Bed Rest Tests Simulating Micro and Lunar Gravity
Source: Front Physiol. 2018 Oct 1;9:1255. doi: 10.3389/fphys.2018.01255 (PMC6174225; doi:10.3389/fphys.2018.01255)
Supplement: TABLE S1 — Acoustic data EXP_type – exp_1 or exp_2, VOLUNTEER_cod – volunteer number, DAY of exp – day of experiment, DAY_cod – cod of the day of experiment, FETa – Forced expiratory noise time, Variability_session – intrasession variability (CV%) of FETa for every person in each day of experiment. [file Table_1.DOC]

|  | **EXP_type** | **VOLUNTEER_cod** | **DAY of exp** | **DAY_cod** | **FETa** | **Variability_session** |
| --- | --- | --- | --- | --- | --- | --- |
| **1** | 1 | 1 | before | 2 | 1.951 |  |
| **2** | 1 | 1 | before | 2 | 1.933 |  |
| **3** | 1 | 1 | before | 2 | 2.055 | 3.3371564 |
| **4** | 1 | 1 | 3 | 3 | 2.324 |  |
| **5** | 1 | 1 | 3 | 3 | 2.211 |  |
| **6** | 1 | 1 | 3 | 3 | 1.986 | 7.89844832 |
| **7** | 1 | 1 | 6 | 4 | 2.111 |  |
| **8** | 1 | 1 | 6 | 4 | 2.269 |  |
| **9** | 1 | 1 | 6 | 4 | 1.584 |  |
| **10** | 1 | 1 | 6 | 4 | 1.983 | 14.7387632 |
| **11** | 1 | 1 | 9 | 5 | 2.205 |  |
| **12** | 1 | 1 | 9 | 5 | 2.664 |  |
| **13** | 1 | 1 | 9 | 5 | 2.757 |  |
| **14** | 1 | 1 | 9 | 5 | 3.132 | 14.1738251 |
| **15** | 1 | 1 | 14 | 6 | 2.772 |  |
| **16** | 1 | 1 | 14 | 6 | 2.051 |  |
| **17** | 1 | 1 | 14 | 6 | 2.468 |  |
| **18** | 1 | 1 | 14 | 6 | 2.566 | 12.3194486 |
| **19** | 1 | 1 | 20 | 7 | 2.671 |  |
| **20** | 1 | 1 | 20 | 7 | 2.265 |  |
| **21** | 1 | 1 | 20 | 7 | 1.870 | 17.6665185 |
| **22** | 1 | 1 | after | 8 | 1.972 |  |
| **23** | 1 | 1 | after | 8 | 1.912 |  |
| **24** | 1 | 1 | after | 8 | 1.721 |  |
| **25** | 1 | 1 | after | 8 | 1.735 |  |
| **26** | 1 | 1 | after | 8 | 1.835 | 5.94493808 |
| **27** | 1 | 2 | before | 2 | 1.061 |  |
| **28** | 1 | 2 | before | 2 | 1.053 |  |
| **29** | 1 | 2 | before | 2 | 1.106 | 2.65340201 |
| **30** | 1 | 2 | 3 | 3 | 1.130 |  |
| **31** | 1 | 2 | 3 | 3 | 1.178 |  |
| **32** | 1 | 2 | 3 | 3 | 1.160 | 2.09365012 |
| **33** | 1 | 2 | 6 | 4 | 1.079 |  |
| **34** | 1 | 2 | 6 | 4 | 1.077 |  |
| **35** | 1 | 2 | 6 | 4 | 1.197 | 6.15726798 |
| **36** | 1 | 2 | 9 | 5 | 1.124 |  |
| **37** | 1 | 2 | 9 | 5 | 1.190 |  |
| **38** | 1 | 2 | 9 | 5 | 1.003 | 8.60372857 |
| **39** | 1 | 2 | 14 | 6 | 1.130 |  |
| **40** | 1 | 2 | 14 | 6 | 1.055 |  |
| **41** | 1 | 2 | 14 | 6 | 1.117 | 3.62854456 |
| **42** | 1 | 2 | 20 | 7 | 1.098 |  |
| **43** | 1 | 2 | 20 | 7 | 1.080 |  |
| **44** | 1 | 2 | 20 | 7 | 1.091 | 0.826667462 |
| **45** | 1 | 2 | after | 8 | 0.956 |  |
| **46** | 1 | 2 | after | 8 | 0.875 |  |
| **47** | 1 | 2 | after | 8 | 0.903 | 4.48073734 |
| **48** | 2 | 3 | before | 2 | 1.071 |  |
| **49** | 2 | 3 | before | 2 | 1.044 |  |
| **50** | 2 | 3 | before | 2 | 1.100 | 2.62596389 |
| **51** | 2 | 3 | 3 | 3 | 1.127 |  |
| **52** | 2 | 3 | 3 | 3 | 1.169 |  |
| **53** | 2 | 3 | 3 | 3 | 1.158 | 1.91309135 |
| **54** | 2 | 3 | 6 | 4 | 1.069 |  |
| **55** | 2 | 3 | 6 | 4 | 1.075 |  |
| **56** | 2 | 3 | 6 | 4 | 1.190 | 6.13030776 |
| **57** | 2 | 3 | 9 | 5 | 1.118 |  |
| **58** | 2 | 3 | 9 | 5 | 1.189 |  |
| **59** | 2 | 3 | 9 | 5 | 1.019 | 7.6700677 |
| **60** | 2 | 3 | 14 | 6 | 1.131 |  |
| **61** | 2 | 3 | 14 | 6 | 1.111 |  |
| **62** | 2 | 3 | 14 | 6 | 1.099 | 1.4536107 |
| **63** | 2 | 3 | 20 | 7 | 1.101 |  |
| **64** | 2 | 3 | 20 | 7 | 1.070 |  |
| **65** | 2 | 3 | 20 | 7 | 1.090 | 1.45807099 |
| **66** | 2 | 3 | after | 8 | 0.997 |  |
| **67** | 2 | 3 | after | 8 | 0.859 |  |
| **68** | 2 | 3 | after | 8 | 0.916 | 7.46351568 |
| **69** | 2 | 4 | before | 2 | 1.884 |  |
| **70** | 2 | 4 | before | 2 | 2.013 |  |
| **71** | 2 | 4 | before | 2 | 1.997 | 3.58925974 |
| **72** | 2 | 4 | 3 | 3 | 2.074 |  |
| **73** | 2 | 4 | 3 | 3 | 2.205 |  |
| **74** | 2 | 4 | 3 | 3 | 2.157 |  |
| **75** | 2 | 4 | 3 | 3 | 2.127 | 2.56242292 |
| **76** | 2 | 4 | 6 | 4 | 2.040 |  |
| **77** | 2 | 4 | 6 | 4 | 2.033 |  |
| **78** | 2 | 4 | 6 | 4 | 2.133 |  |
| **79** | 2 | 4 | 6 | 4 | 2.319 | 6.24514508 |
| **80** | 2 | 4 | 9 | 5 | 2.001 |  |
| **81** | 2 | 4 | 9 | 5 | 2.176 |  |
| **82** | 2 | 4 | 9 | 5 | 1.909 |  |
| **83** | 2 | 4 | 9 | 5 | 1.455 |  |
| **84** | 2 | 4 | 9 | 5 | 1.946 | 14.1050314 |
| **85** | 2 | 4 | 14 | 6 | 2.488 |  |
| **86** | 2 | 4 | 14 | 6 | 2.235 |  |
| **87** | 2 | 4 | 14 | 6 | 2.046 |  |
| **88** | 2 | 4 | 14 | 6 | 2.046 | 9.50937358 |
| **89** | 2 | 4 | 20 | 7 | 2.212 |  |
| **90** | 2 | 4 | 20 | 7 | 1.950 |  |
| **91** | 2 | 4 | 20 | 7 | 1.926 |  |
| **92** | 2 | 4 | 20 | 7 | 2.400 |  |
| **93** | 2 | 4 | 20 | 7 | 2.406 | 10.7188359 |
| **94** | 2 | 4 | after | 8 | 2.040 |  |
| **95** | 2 | 4 | after | 8 | 2.074 |  |
| **96** | 2 | 4 | after | 8 | 2.263 |  |
| **97** | 2 | 4 | after | 8 | 2.115 |  |
| **98** | 2 | 4 | after | 8 | 1.946 | 5.56718856 |
| **99** | 2 | 5 | before | 2 | 1.134 |  |
| **100** | 2 | 5 | before | 2 | 1.114 |  |
| **101** | 2 | 5 | before | 2 | 1.109 | 1.17577439 |
| **102** | 2 | 5 | 3 | 3 | 1.341 |  |
| **103** | 2 | 5 | 3 | 3 | 1.253 |  |
| **104** | 2 | 5 | 3 | 3 | 1.253 |  |
| **105** | 2 | 5 | 3 | 3 | 1.143 | 6.49721311 |
| **106** | 2 | 5 | 6 | 4 | 1.063 |  |
| **107** | 2 | 5 | 6 | 4 | 1.027 |  |
| **108** | 2 | 5 | 6 | 4 | 1.142 | 5.43593389 |
| **109** | 2 | 5 | 9 | 5 | 1.126 |  |
| **110** | 2 | 5 | 9 | 5 | 1.119 |  |
| **111** | 2 | 5 | 9 | 5 | 1.066 | 2.95527242 |
| **112** | 2 | 5 | 14 | 6 | 1.079 |  |
| **113** | 2 | 5 | 14 | 6 | 1.105 |  |
| **114** | 2 | 5 | 14 | 6 | 1.107 |  |
| **115** | 2 | 5 | 14 | 6 | 1.128 | 1.78482678 |
| **116** | 2 | 5 | 20 | 7 | 1.053 |  |
| **117** | 2 | 5 | 20 | 7 | 1.163 |  |
| **118** | 2 | 5 | 20 | 7 | 1.008 |  |
| **119** | 2 | 5 | 20 | 7 | 1.207 | 8.37114655 |
| **120** | 2 | 5 | after | 8 | 1.018 |  |
| **121** | 2 | 5 | after | 8 | 1.089 |  |
| **122** | 2 | 5 | after | 8 | 1.120 |  |
| **123** | 2 | 5 | after | 8 | 1.103 | 4.14330898 |
| **124** | 1 | 6 | before | 2 | 1.139 |  |
| **125** | 1 | 6 | before | 2 | 1.206 |  |
| **126** | 1 | 6 | before | 2 | 1.183 |  |
| **127** | 1 | 6 | before | 2 | 1.175 | 2.34537503 |
| **128** | 1 | 6 | 3 | 3 | 1.147 |  |
| **129** | 1 | 6 | 3 | 3 | 1.324 |  |
| **130** | 1 | 6 | 3 | 3 | 1.224 |  |
| **131** | 1 | 6 | 3 | 3 | 1.276 | 6.08588745 |
| **132** | 1 | 6 | 6 | 4 | 1.437 |  |
| **133** | 1 | 6 | 6 | 4 | 1.351 |  |
| **134** | 1 | 6 | 6 | 4 | 1.367 |  |
| **135** | 1 | 6 | 6 | 4 | 1.503 | 4.92424116 |
| **136** | 1 | 6 | 9 | 5 | 1.279 |  |
| **137** | 1 | 6 | 9 | 5 | 1.276 |  |
| **138** | 1 | 6 | 9 | 5 | 1.454 |  |
| **139** | 1 | 6 | 9 | 5 | 1.371 | 6.34422827 |
| **140** | 1 | 6 | 14 | 6 | 1.571 |  |
| **141** | 1 | 6 | 14 | 6 | 1.606 |  |
| **142** | 1 | 6 | 14 | 6 | 1.462 |  |
| **143** | 1 | 6 | 14 | 6 | 1.418 | 5.86070821 |
| **144** | 1 | 6 | 20 | 7 | 1.393 |  |
| **145** | 1 | 6 | 20 | 7 | 1.250 |  |
| **146** | 1 | 6 | 20 | 7 | 1.347 |  |
| **147** | 1 | 6 | 20 | 7 | 1.259 | 5.30377057 |
| **148** | 1 | 6 | after | 8 | 1.502 |  |
| **149** | 1 | 6 | after | 8 | 1.352 |  |
| **150** | 1 | 6 | after | 8 | 1.505 |  |
| **151** | 1 | 6 | after | 8 | 1.208 | 10.2148304 |
| **152** | 1 | 7 | before | 2 | 2.038 |  |
| **153** | 1 | 7 | before | 2 | 1.857 |  |
| **154** | 1 | 7 | before | 2 | 2.088 | 6.09698195 |
| **155** | 1 | 7 | 3 | 3 | 2.398 |  |
| **156** | 1 | 7 | 3 | 3 | 2.322 |  |
| **157** | 1 | 7 | 3 | 3 | 2.131 | 6.02660091 |
| **158** | 1 | 7 | 6 | 4 | 2.644 |  |
| **159** | 1 | 7 | 6 | 4 | 2.374 |  |
| **160** | 1 | 7 | 6 | 4 | 2.455 | 5.55781095 |
| **161** | 1 | 7 | 9 | 5 | 2.552 |  |
| **162** | 1 | 7 | 9 | 5 | 2.545 |  |
| **163** | 1 | 7 | 9 | 5 | 2.174 |  |
| **164** | 1 | 7 | 9 | 5 | 2.224 | 8.54254414 |
| **165** | 1 | 7 | 14 | 6 | 2.747 |  |
| **166** | 1 | 7 | 14 | 6 | 2.434 |  |
| **167** | 1 | 7 | 14 | 6 | 2.726 |  |
| **168** | 1 | 7 | 14 | 6 | 2.350 | 7.88485835 |
| **169** | 1 | 7 | 20 | 7 | 2.512 |  |
| **170** | 1 | 7 | 20 | 7 | 2.765 |  |
| **171** | 1 | 7 | 20 | 7 | 2.610 | 4.84553878 |
| **172** | 1 | 7 | after | 8 | 1.926 |  |
| **173** | 1 | 7 | after | 8 | 1.849 |  |
| **174** | 1 | 7 | after | 8 | 1.998 |  |
| **175** | 1 | 7 | after | 8 | 2.233 | 8.26833593 |
| **176** | 2 | 8 | before | 2 | 1.566 |  |
| **177** | 2 | 8 | before | 2 | 1.519 |  |
| **178** | 2 | 8 | before | 2 | 1.515 | 1.85188165 |
| **179** | 2 | 8 | 3 | 3 | 1.440 |  |
| **180** | 2 | 8 | 3 | 3 | 1.865 |  |
| **181** | 2 | 8 | 3 | 3 | 1.521 | 14.0320044 |
| **182** | 2 | 8 | 6 | 4 | 1.610 |  |
| **183** | 2 | 8 | 6 | 4 | 1.746 |  |
| **184** | 2 | 8 | 6 | 4 | 1.517 | 7.11341419 |
| **185** | 2 | 8 | 9 | 5 | 1.494 |  |
| **186** | 2 | 8 | 9 | 5 | 1.580 |  |
| **187** | 2 | 8 | 9 | 5 | 1.300 |  |
| **188** | 2 | 8 | 9 | 5 | 1.495 |  |
| **189** | 2 | 8 | 9 | 5 | 1.507 | 7.06774695 |
| **190** | 2 | 8 | 14 | 6 | 1.359 |  |
| **191** | 2 | 8 | 14 | 6 | 1.475 |  |
| **192** | 2 | 8 | 14 | 6 | 1.219 | 9.49099622 |
| **193** | 2 | 8 | 20 | 7 | 1.332 |  |
| **194** | 2 | 8 | 20 | 7 | 1.396 |  |
| **195** | 2 | 8 | 20 | 7 | 1.437 |  |
| **196** | 2 | 8 | 20 | 7 | 1.463 |  |
| **197** | 2 | 8 | 20 | 7 | 1.622 | 7.46734825 |
| **198** | 2 | 8 | after | 8 | 1.132 |  |
| **199** | 2 | 8 | after | 8 | 1.640 |  |
| **200** | 2 | 8 | after | 8 | 1.112 |  |
| **201** | 2 | 8 | after | 8 | 1.122 |  |
| **202** | 2 | 8 | after | 8 | 1.080 | 19.4827665 |
| **203** | 2 | 9 | before | 2 | 1.130 |  |
| **204** | 2 | 9 | before | 2 | 1.187 |  |
| **205** | 2 | 9 | before | 2 | 1.078 |  |
| **206** | 2 | 9 | before | 2 | 1.285 | 7.56182074 |
| **207** | 2 | 9 | 3 | 3 | 1.428 |  |
| **208** | 2 | 9 | 3 | 3 | 1.281 |  |
| **209** | 2 | 9 | 3 | 3 | 1.529 |  |
| **210** | 2 | 9 | 3 | 3 | 1.223 | 10.1711118 |
| **211** | 2 | 9 | 6 | 4 | 1.264 |  |
| **212** | 2 | 9 | 6 | 4 | 1.205 |  |
| **213** | 2 | 9 | 6 | 4 | 1.357 |  |
| **214** | 2 | 9 | 6 | 4 | 1.185 | 6.160121 |
| **215** | 2 | 9 | 9 | 5 | 1.193 |  |
| **216** | 2 | 9 | 9 | 5 | 1.193 |  |
| **217** | 2 | 9 | 9 | 5 | 1.209 |  |
| **218** | 2 | 9 | 9 | 5 | 1.157 |  |
| **219** | 2 | 9 | 9 | 5 | 1.136 | 2.56402483 |
| **220** | 2 | 9 | 14 | 6 | 1.247 |  |
| **221** | 2 | 9 | 14 | 6 | 1.337 |  |
| **222** | 2 | 9 | 14 | 6 | 1.183 |  |
| **223** | 2 | 9 | 14 | 6 | 1.084 | 8.79060833 |
| **224** | 2 | 9 | 20 | 7 | 1.262 |  |
| **225** | 2 | 9 | 20 | 7 | 1.186 |  |
| **226** | 2 | 9 | 20 | 7 | 1.191 |  |
| **227** | 2 | 9 | 20 | 7 | 1.192 | 3.01771947 |
| **228** | 2 | 9 | after | 8 | 1.244 |  |
| **229** | 2 | 9 | after | 8 | 1.393 |  |
| **230** | 2 | 9 | after | 8 | 1.281 |  |
| **231** | 2 | 9 | after | 8 | 1.321 |  |
| **232** | 2 | 9 | after | 8 | 1.337 | 4.32089365 |
| **233** | 2 | 10 | before | 2 | 1.434 |  |
| **234** | 2 | 10 | before | 2 | 1.507 |  |
| **235** | 2 | 10 | before | 2 | 1.368 | 4.84503664 |
| **236** | 2 | 10 | 3 | 3 | 2.023 |  |
| **237** | 2 | 10 | 3 | 3 | 2.097 |  |
| **238** | 2 | 10 | 3 | 3 | 2.126 | 2.52782818 |
| **239** | 2 | 10 | 6 | 4 | 1.904 |  |
| **240** | 2 | 10 | 6 | 4 | 1.829 |  |
| **241** | 2 | 10 | 6 | 4 | 1.906 |  |
| **242** | 2 | 10 | 6 | 4 | 1.902 | 1.97563037 |
| **243** | 2 | 10 | 9 | 5 | 2.107 |  |
| **244** | 2 | 10 | 9 | 5 | 1.976 |  |
| **245** | 2 | 10 | 9 | 5 | 1.937 |  |
| **246** | 2 | 10 | 9 | 5 | 2.072 | 3.92737183 |
| **247** | 2 | 10 | 14 | 6 | 1.998 |  |
| **248** | 2 | 10 | 14 | 6 | 2.381 |  |
| **249** | 2 | 10 | 14 | 6 | 1.963 | 10.9856508 |
| **250** | 2 | 10 | 20 | 7 | 1.882 |  |
| **251** | 2 | 10 | 20 | 7 | 1.788 |  |
| **252** | 2 | 10 | 20 | 7 | 1.911 |  |
| **253** | 2 | 10 | 20 | 7 | 1.954 | 3.74160695 |
| **254** | 2 | 10 | after | 8 | 1.496 |  |
| **255** | 2 | 10 | after | 8 | 1.482 |  |
| **256** | 2 | 10 | after | 8 | 1.477 |  |
| **257** | 2 | 10 | after | 8 | 1.432 | 1.86012753 |
| **258** | 1 | 11 | before | 2 | 1.041 |  |
| **259** | 1 | 11 | before | 2 | 0.950 |  |
| **260** | 1 | 11 | before | 2 | 1.054 | 5.55434601 |
| **261** | 1 | 11 | 3 | 3 | 1.105 |  |
| **262** | 1 | 11 | 3 | 3 | 1.099 |  |
| **263** | 1 | 11 | 3 | 3 | 1.103 | 0.27341737 |
| **264** | 1 | 11 | 6 | 4 | 1.084 |  |
| **265** | 1 | 11 | 6 | 4 | 1.031 |  |
| **266** | 1 | 11 | 6 | 4 | 1.077 | 2.73822144 |
| **267** | 1 | 11 | 9 | 5 | 1.231 |  |
| **268** | 1 | 11 | 9 | 5 | 1.046 |  |
| **269** | 1 | 11 | 9 | 5 | 1.013 | 10.7290385 |
| **270** | 1 | 11 | 14 | 6 | 1.088 |  |
| **271** | 1 | 11 | 14 | 6 | 1.067 |  |
| **272** | 1 | 11 | 14 | 6 | 1.019 | 3.37668355 |
| **273** | 1 | 11 | 20 | 7 | 1.197 |  |
| **274** | 1 | 11 | 20 | 7 | 1.637 |  |
| **275** | 1 | 11 | 20 | 7 | 1.252 | 17.6063016 |
| **276** | 1 | 11 | after | 8 | 1.139 |  |
| **277** | 1 | 11 | after | 8 | 1.334 |  |
| **278** | 1 | 11 | after | 8 | 1.074 | 11.4543861 |
