# Supplementary material for: A Technique of Forced Expiratory Noise Time Evaluation Provides Distinguishing Human Pulmonary Ventilation Dynamics During Long-Term Head-Down and Head-Up Tilt Bed Rest Tests Simulating Micro and Lunar Gravity
Source: Front Physiol. 2018 Oct 1;9:1255. doi: 10.3389/fphys.2018.01255 (PMC6174225; doi:10.3389/fphys.2018.01255)
Supplement: TABLE S2 — Spirometry data EXP_type_SP – exp_1 or exp_2 for spirometry, VOLUNTEER_cod_SP – volunteer number for spirometry, DAY_exp_SP – day of experiment for spirometry, DAY_SP_cod – cod of the day of experiment for spirometry, FEV1 – FEV1, FVC – FVC, FEV1/FVC – FEV1/FVC MMEF – MMEF, VC – VC. [file Table_2.DOC]

|  | **EXP_type_SP** | **VOLUNTEER_cod_SP** | **DAY_exp_SP** | **DAY_SP_cod** | **FEV1** | **FVC** | **FEV1/FVC** | **MMEF** | **VC** |
| --- | --- | --- | --- | --- | --- | --- | --- | --- | --- |
| **1** | 1 | 1 | before | 2 | 4.360 | 5.460 | 0.799 | 3.860 | 5.340 |
| **2** | 1 | 1 | before | 2 | 4.280 | 5.390 | 0.794 | 3.720 | 5.230 |
| **3** | 1 | 1 | beore | 2 | 4.290 | 5.400 | 0.794 | 3.610 | 5.290 |
| **4** | 1 | 1 | before | 2 | 4.210 | 5.450 | 0.772 | 3.440 |  |
| **5** | 1 | 1 | 3 | 3 | 4.280 | 5.500 | 0.778 | 3.910 | 5.250 |
| **6** | 1 | 1 | 3 | 3 | 4.160 | 5.330 | 0.780 | 3.290 | 5.100 |
| **7** | 1 | 1 | 3 | 3 | 4.250 | 5.430 | 0.783 | 3.540 | 5.090 |
| **8** | 1 | 1 | 3 | 3 | 4.180 | 5.450 | 0.767 | 3.610 | 5.210 |
| **9** | 1 | 1 | 3 | 3 | 4.220 | 5.280 | 0.799 | 3.400 |  |
| **10** | 1 | 1 | 6 | 4 | 4.310 | 5.470 | 0.788 | 3.610 | 5.330 |
| **11** | 1 | 1 | 6 | 4 | 4.260 | 5.450 | 0.782 | 3.760 | 5.210 |
| **12** | 1 | 1 | 6 | 4 | 4.240 | 5.470 | 0.775 | 3.530 | 5.290 |
| **13** | 1 | 1 | 6 | 4 | 4.220 | 5.470 | 0.771 | 3.480 | 5.330 |
| **14** | 1 | 1 | 6 | 4 | 4.250 | 5.440 | 0.781 | 3.590 |  |
| **15** | 1 | 1 | 9 | 5 | 4.240 | 5.570 | 0.761 | 3.570 | 5.430 |
| **16** | 1 | 1 | 9 | 5 | 4.220 | 5.400 | 0.781 | 3.220 | 5.330 |
| **17** | 1 | 1 | 9 | 5 | 4.170 | 5.490 | 0.760 | 3.290 | 5.380 |
| **18** | 1 | 1 | 14 | 6 | 4.270 | 5.650 | 0.756 | 3.400 | 5.500 |
| **19** | 1 | 1 | 14 | 6 | 4.140 | 5.420 | 0.764 | 3.290 | 5.470 |
| **20** | 1 | 1 | 14 | 6 | 4.260 | 5.550 | 0.768 | 3.430 | 5.400 |
| **21** | 1 | 1 | 14 | 6 | 4.250 | 5.530 | 0.769 |  | 5.480 |
| **22** | 1 | 1 | 20 | 7 | 4.340 | 5.660 | 0.767 | 3.410 | 5.480 |
| **23** | 1 | 1 | 20 | 7 | 4.180 | 5.420 | 0.771 | 3.870 | 5.360 |
| **24** | 1 | 1 | 20 | 7 | 4.160 | 5.470 | 0.761 | 3.110 | 5.460 |
| **25** | 1 | 1 | 20 | 7 | 4.300 | 5.550 | 0.775 | 3.470 | 5.420 |
| **26** | 1 | 1 | 20 | 7 | 4.330 | 5.610 | 0.772 | 3.420 |  |
| **27** | 1 | 1 | after | 8 | 4.600 | 5.800 | 0.793 | 4.250 | 5.800 |
| **28** | 1 | 1 | after | 8 | 4.470 | 5.490 | 0.814 | 4.270 | 5.760 |
| **29** | 1 | 1 | after | 8 | 4.550 | 5.720 | 0.795 | 3.920 | 5.610 |
| **30** | 1 | 1 | after | 8 | 4.600 | 5.790 | 0.794 | 4.120 | 5.760 |
| **31** | 1 | 1 | after | 8 | 4.550 | 5.780 | 0.787 | 3.930 |  |
| **32** | 1 | 2 | before | 2 | 3.450 | 3.750 | 0.920 | 3.400 | 3.710 |
| **33** | 1 | 2 | before | 2 | 3.370 | 3.670 | 0.918 | 3.520 | 3.540 |
| **34** | 1 | 2 | befor | 2 | 3.410 | 3.700 | 0.922 | 3.500 | 3.660 |
| **35** | 1 | 2 | 3 | 3 | 3.290 | 3.580 | 0.919 | 3.200 | 3.620 |
| **36** | 1 | 2 | 3 | 3 | 3.220 | 3.550 | 0.907 | 3.220 | 3.470 |
| **37** | 1 | 2 | 3 | 3 | 3.250 | 3.370 | 0.964 | 3.320 | 3.520 |
| **38** | 1 | 2 | 3 | 3 | 3.290 | 3.540 | 0.929 | 3.340 | 3.450 |
| **39** | 1 | 2 | 6 | 4 | 3.370 | 3.650 | 0.923 | 3.340 | 3.640 |
| **40** | 1 | 2 | 6 | 4 | 3.340 | 3.620 | 0.923 | 3.370 | 3.490 |
| **41** | 1 | 2 | 6 | 4 | 3.300 | 3.590 | 0.919 | 3.400 | 3.560 |
| **42** | 1 | 2 | 6 | 4 | 3.350 | 3.590 | 0.933 | 3.490 | 3.610 |
| **43** | 1 | 2 | 9 | 5 | 3.440 | 3.700 | 0.930 | 3.530 | 3.730 |
| **44** | 1 | 2 | 9 | 5 | 3.420 | 3.630 | 0.942 | 3.450 | 3.700 |
| **45** | 1 | 2 | 9 | 5 | 3.410 | 3.690 | 0.924 | 3.490 | 3.720 |
| **46** | 1 | 2 | 9 | 5 | 3.430 | 3.690 | 0.930 | 3.510 | 3.620 |
| **47** | 1 | 2 | 14 | 6 | 3.450 | 3.710 | 0.930 | 3.520 | 3.690 |
| **48** | 1 | 2 | 14 | 6 | 3.440 | 3.700 | 0.930 | 3.510 | 3.600 |
| **49** | 1 | 2 | 14 | 6 | 3.410 | 3.700 | 0.922 | 3.610 | 3.640 |
| **50** | 1 | 2 | 14 | 6 | 3.370 | 3.620 | 0.931 | 3.500 | 3.680 |
| **51** | 1 | 2 | 14 | 6 | 3.450 | 3.610 | 0.956 | 3.490 |  |
| **52** | 1 | 2 | 20 | 7 | 3.480 | 3.810 | 0.913 | 3.360 | 3.770 |
| **53** | 1 | 2 | 20 | 7 | 3.450 | 3.720 | 0.927 | 3.400 | 3.660 |
| **54** | 1 | 2 | 20 | 7 | 3.420 | 3.760 | 0.910 | 3.520 | 3.690 |
| **55** | 1 | 2 | 20 | 7 | 3.470 | 3.740 | 0.928 | 3.540 | 3.600 |
| **56** | 1 | 2 | 20 | 7 | 3.430 | 3.750 | 0.915 | 3.440 | 3.640 |
| **57** | 1 | 2 | after | 8 | 3.580 | 3.800 | 0.942 | 3.990 | 3.750 |
| **58** | 1 | 2 | after | 8 | 3.430 | 3.740 | 0.917 | 3.890 | 3.670 |
| **59** | 1 | 2 | after | 8 | 3.470 | 3.680 | 0.943 | 3.970 | 3.670 |
| **60** | 1 | 2 | after | 8 | 3.550 | 3.720 | 0.954 | 3.810 | 3.660 |
| **61** | 2 | 3 | before | 2 | 4.380 | 5.560 | 0.788 | 4.050 | 5.440 |
| **62** | 2 | 3 | before | 2 | 4.220 | 5.190 | 0.813 | 3.850 | 5.290 |
| **63** | 2 | 3 | before | 2 | 4.250 | 5.310 | 0.800 | 3.660 | 5.280 |
| **64** | 2 | 3 | before | 2 | 4.240 | 5.420 | 0.782 | 3.840 | 5.370 |
| **65** | 2 | 3 | 3 | 3 | 4.160 | 5.350 | 0.778 | 3.100 | 5.310 |
| **66** | 2 | 3 | 3 | 3 | 3.860 | 5.030 | 0.767 | 3.160 | 5.310 |
| **67** | 2 | 3 | 3 | 3 | 3.990 | 5.230 | 0.763 | 3.300 | 5.220 |
| **68** | 2 | 3 | 3 | 3 | 4.050 | 5.290 | 0.766 | 3.480 |  |
| **69** | 2 | 3 | 6 | 4 | 3.870 | 5.080 | 0.762 | 3.170 | 5.240 |
| **70** | 2 | 3 | 6 | 4 | 3.740 | 5.020 | 0.745 | 2.800 | 5.130 |
| **71** | 2 | 3 | 6 | 4 | 3.870 | 5.010 | 0.772 | 3.120 | 5.140 |
| **72** | 2 | 3 | 6 | 4 | 3.830 | 5.050 | 0.758 | 2.960 |  |
| **73** | 2 | 3 | 9 | 5 | 4.010 | 5.300 | 0.757 | 3.070 | 5.500 |
| **74** | 2 | 3 | 9 | 5 | 3.950 | 5.220 | 0.757 | 2.990 | 5.490 |
| **75** | 2 | 3 | 9 | 5 | 3.960 | 5.290 | 0.749 | 2.710 | 5.310 |
| **76** | 2 | 3 | 9 | 5 | 3.900 | 5.290 | 0.737 | 3.160 | 5.320 |
| **77** | 2 | 3 | 14 | 6 | 4.160 | 5.550 | 0.750 | 3.410 | 5.460 |
| **78** | 2 | 3 | 14 | 6 | 4.120 | 5.330 | 0.773 | 3.030 | 5.400 |
| **79** | 2 | 3 | 14 | 6 | 4.000 | 5.320 | 0.752 | 2.740 | 5.400 |
| **80** | 2 | 3 | 14 | 6 | 4.040 | 5.460 | 0.740 | 3.280 |  |
| **81** | 2 | 3 | 20 | 7 | 4.070 | 5.330 | 0.764 | 3.210 | 5.250 |
| **82** | 2 | 3 | 20 | 7 | 4.030 | 5.280 | 0.763 | 3.040 | 5.240 |
| **83** | 2 | 3 | 20 | 7 | 3.950 | 5.230 | 0.755 | 3.250 | 5.220 |
| **84** | 2 | 3 | 20 | 7 | 4.070 | 5.320 | 0.765 | 3.190 |  |
| **85** | 2 | 3 | after | 8 | 4.350 | 5.440 | 0.800 | 3.900 | 5.460 |
| **86** | 2 | 3 | after | 8 | 4.220 | 5.280 | 0.799 | 3.950 | 5.400 |
| **87** | 2 | 3 | after | 8 | 4.330 | 5.350 | 0.809 | 4.110 | 5.430 |
| **88** | 2 | 3 | after | 8 | 4.310 | 5.410 | 0.797 | 4.010 |  |
| **89** | 2 | 4 | before | 2 | 4.490 | 6.050 | 0.742 | 3.340 | 5.890 |
| **90** | 2 | 4 | before | 2 | 4.440 | 6.030 | 0.736 | 3.450 | 5.700 |
| **91** | 2 | 4 | before | 2 | 4.360 | 5.850 | 0.745 | 3.340 | 5.740 |
| **92** | 2 | 4 | before | 2 | 4.280 | 5.620 | 0.762 | 3.410 | 5.820 |
| **93** | 2 | 4 | before | 2 | 4.320 | 5.830 | 0.741 | 3.300 |  |
| **94** | 2 | 4 | 3 | 3 | 4.230 | 5.980 | 0.707 | 2.980 | 5.640 |
| **95** | 2 | 4 | 3 | 3 | 4.090 | 5.670 | 0.721 | 2.910 | 5.470 |
| **96** | 2 | 4 | 3 | 3 | 4.230 | 5.760 | 0.734 | 3.160 | 5.470 |
| **97** | 2 | 4 | 3 | 3 | 4.140 | 5.930 | 0.698 | 2.790 | 5.520 |
| **98** | 2 | 4 | 3 | 3 | 4.160 | 5.830 | 0.714 | 2.940 |  |
| **99** | 2 | 4 | 6 | 4 | 4.290 | 5.970 | 0.719 | 3.140 | 5.750 |
| **100** | 2 | 4 | 6 | 4 | 4.260 | 5.850 | 0.728 | 3.370 | 5.610 |
| **101** | 2 | 4 | 6 | 4 | 4.240 | 5.720 | 0.741 | 3.030 | 5.750 |
| **102** | 2 | 4 | 6 | 4 | 4.290 | 5.880 | 0.730 | 3.210 |  |
| **103** | 2 | 4 | 6 | 4 | 4.250 | 5.960 | 0.713 | 3.010 |  |
| **104** | 2 | 4 | 9 | 5 | 4.460 | 5.880 | 0.759 | 3.350 | 5.640 |
| **105** | 2 | 4 | 9 | 5 | 4.360 | 5.850 | 0.745 | 3.330 | 5.600 |
| **106** | 2 | 4 | 9 | 5 | 4.350 | 5.880 | 0.740 | 3.430 | 5.560 |
| **107** | 2 | 4 | 9 | 5 | 4.410 | 5.880 | 0.750 | 3.570 |  |
| **108** | 2 | 4 | 14 | 6 | 4.500 | 5.970 | 0.754 | 3.560 | 5.700 |
| **109** | 2 | 4 | 14 | 6 | 4.400 | 5.950 | 0.739 | 3.350 | 5.590 |
| **110** | 2 | 4 | 14 | 6 | 4.320 | 5.900 | 0.732 | 3.240 | 5.660 |
| **111** | 2 | 4 | 14 | 6 | 4.410 | 5.890 | 0.749 | 3.470 |  |
| **112** | 2 | 4 | 20 | 7 | 4.510 | 6.040 | 0.747 | 3.270 | 5.870 |
| **113** | 2 | 4 | 20 | 7 | 4.400 | 6.010 | 0.732 | 3.360 | 5.690 |
| **114** | 2 | 4 | 20 | 7 | 4.440 | 6.030 | 0.736 | 3.500 | 5.780 |
| **115** | 2 | 4 | 20 | 7 | 4.360 | 6.020 | 0.724 | 3.210 | 5.700 |
| **116** | 2 | 4 | after | 8 | 4.620 | 5.980 | 0.773 | 3.830 | 5.960 |
| **117** | 2 | 4 | after | 8 | 4.590 | 5.960 | 0.770 | 3.770 | 5.860 |
| **118** | 2 | 4 | after | 8 | 4.620 | 5.940 | 0.778 | 3.850 | 5.900 |
| **119** | 2 | 4 | after | 8 | 4.590 | 5.980 | 0.768 | 3.760 |  |
| **120** | 2 | 5 | before | 2 | 5.160 | 5.400 | 0.956 | 5.560 | 5.290 |
| **121** | 2 | 5 | before | 2 | 5.090 | 5.360 | 0.950 | 5.460 | 5.270 |
| **122** | 2 | 5 | before | 2 | 5.100 | 5.260 | 0.970 | 5.560 | 5.190 |
| **123** | 2 | 5 | before | 2 | 5.130 | 5.360 | 0.957 | 5.480 |  |
| **124** | 2 | 5 | 3 | 3 | 5.010 | 5.380 | 0.931 | 5.450 | 5.150 |
| **125** | 2 | 5 | 3 | 3 | 4.910 | 5.300 | 0.926 | 5.190 | 5.190 |
| **126** | 2 | 5 | 3 | 3 | 4.910 | 5.240 | 0.937 | 5.220 | 4.990 |
| **127** | 2 | 5 | 3 | 3 | 4.840 | 5.230 | 0.925 | 5.140 |  |
| **128** | 2 | 5 | 6 | 4 | 4.930 | 5.250 | 0.939 | 5.240 | 5.350 |
| **129** | 2 | 5 | 6 | 4 | 4.830 | 5.150 | 0.938 | 5.250 | 5.230 |
| **130** | 2 | 5 | 6 | 4 | 4.840 | 5.200 | 0.931 | 5.280 | 5.170 |
| **131** | 2 | 5 | 6 | 4 | 4.860 | 5.200 | 0.935 | 5.250 | 5.290 |
| **132** | 2 | 5 | 9 | 5 | 5.000 | 5.330 | 0.938 | 5.410 | 5.190 |
| **133** | 2 | 5 | 9 | 5 | 4.970 | 5.240 | 0.948 | 5.370 | 5.090 |
| **134** | 2 | 5 | 9 | 5 | 5.000 | 5.280 | 0.947 | 5.430 | 5.180 |
| **135** | 2 | 5 | 9 | 5 | 4.890 | 5.140 | 0.951 | 5.330 |  |
| **136** | 2 | 5 | 14 | 6 | 5.140 | 5.420 | 0.948 | 5.510 | 5.330 |
| **137** | 2 | 5 | 14 | 6 | 5.100 | 5.410 | 0.943 | 5.400 | 5.230 |
| **138** | 2 | 5 | 14 | 6 | 5.080 | 5.360 | 0.948 | 5.460 | 5.270 |
| **139** | 2 | 5 | 14 | 6 | 5.050 | 5.410 | 0.933 | 5.160 | 5.260 |
| **140** | 2 | 5 | 20 | 7 | 5.140 | 5.450 | 0.943 | 5.460 | 5.490 |
| **141** | 2 | 5 | 20 | 7 | 5.130 | 5.420 | 0.946 | 5.380 | 5.330 |
| **142** | 2 | 5 | 20 | 7 | 5.110 | 5.450 | 0.938 | 5.410 | 5.350 |
| **143** | 2 | 5 | 20 | 7 | 5.100 | 5.370 | 0.950 | 5.400 |  |
| **144** | 2 | 5 | after | 8 | 5.080 | 5.360 | 0.948 | 5.530 | 5.250 |
| **145** | 2 | 5 | after | 8 | 5.050 | 5.240 | 0.964 | 5.480 | 5.190 |
| **146** | 2 | 5 | after | 8 | 5.010 | 5.230 | 0.958 | 5.490 | 5.250 |
| **147** | 2 | 5 | after | 8 | 5.020 | 5.250 | 0.956 | 5.370 |  |
| **148** | 1 | 6 | before | 2 | 5.550 | 6.390 | 0.869 | 6.370 | 6.300 |
| **149** | 1 | 6 | before | 2 | 5.370 | 6.330 | 0.848 | 6.760 | 6.200 |
| **150** | 1 | 6 | before | 2 | 5.540 | 6.230 | 0.889 | 6.450 | 6.260 |
| **151** | 1 | 6 | before | 2 | 5.450 | 6.270 | 0.869 | 6.550 |  |
| **152** | 1 | 6 | before | 2 | 5.460 | 6.160 | 0.886 | 6.510 |  |
| **153** | 1 | 6 | 3 | 3 | 5.380 | 6.200 | 0.868 | 6.290 | 6.110 |
| **154** | 1 | 6 | 3 | 3 | 5.240 | 6.040 | 0.868 | 6.390 | 6.040 |
| **155** | 1 | 6 | 3 | 3 | 5.290 | 6.120 | 0.864 | 6.130 | 6.020 |
| **156** | 1 | 6 | 3 | 3 | 5.230 | 6.180 | 0.846 | 5.590 |  |
| **157** | 1 | 6 | 6 | 4 | 5.250 | 6.160 | 0.852 | 6.170 | 6.010 |
| **158** | 1 | 6 | 6 | 4 | 5.060 | 6.100 | 0.830 | 5.630 | 5.980 |
| **159** | 1 | 6 | 6 | 4 | 5.250 | 6.040 | 0.869 | 5.860 | 5.940 |
| **160** | 1 | 6 | 6 | 4 | 5.090 | 6.110 | 0.833 | 5.500 |  |
| **161** | 1 | 6 | 6 | 4 | 5.140 | 6.050 | 0.850 | 5.550 |  |
| **162** | 1 | 6 | 9 | 5 | 5.280 | 6.130 | 0.861 | 6.230 | 6.120 |
| **163** | 1 | 6 | 9 | 5 | 5.160 | 6.050 | 0.853 | 5.710 | 6.080 |
| **164** | 1 | 6 | 9 | 5 | 5.150 | 6.080 | 0.847 | 5.890 | 6.020 |
| **165** | 1 | 6 | 9 | 5 | 5.250 | 6.020 | 0.872 | 6.030 |  |
| **166** | 1 | 6 | 14 | 6 | 5.190 | 6.000 | 0.865 | 5.750 | 6.090 |
| **167** | 1 | 6 | 14 | 6 | 5.060 | 5.910 | 0.856 | 5.840 | 6.000 |
| **168** | 1 | 6 | 14 | 6 | 5.030 | 5.850 | 0.860 | 5.700 | 5.980 |
| **169** | 1 | 6 | 14 | 6 | 4.980 | 5.830 | 0.854 | 6.100 | 6.010 |
| **170** | 1 | 6 | 20 | 7 | 5.310 | 6.140 | 0.865 | 6.050 | 6.070 |
| **171** | 1 | 6 | 20 | 7 | 5.120 | 5.940 | 0.862 | 6.320 | 5.940 |
| **172** | 1 | 6 | 20 | 7 | 5.070 | 6.100 | 0.831 | 6.230 | 5.990 |
| **173** | 1 | 6 | 20 | 7 | 5.240 | 5.860 | 0.894 | 6.080 | 6.030 |
| **174** | 1 | 6 | 20 | 7 | 5.310 | 6.080 | 0.873 | 6.280 |  |
| **175** | 1 | 6 | after | 8 | 5.520 | 6.170 | 0.895 | 6.660 | 6.160 |
| **176** | 1 | 6 | after | 8 | 5.500 | 6.100 | 0.902 | 6.520 | 6.020 |
| **177** | 1 | 6 | after | 8 | 5.430 | 6.160 | 0.881 | 6.700 | 6.100 |
| **178** | 1 | 6 | after | 8 | 5.480 | 6.170 | 0.888 | 6.860 | 6.060 |
| **179** | 1 | 7 | before | 2 | 4.680 | 6.510 | 0.719 | 3.660 | 6.460 |
| **180** | 1 | 7 | before | 2 | 4.580 | 6.170 | 0.742 | 3.580 | 6.380 |
| **181** | 1 | 7 | before | 2 | 4.660 | 6.390 | 0.729 | 3.630 | 6.440 |
| **182** | 1 | 7 | before | 2 | 4.620 | 6.380 | 0.724 | 3.550 |  |
| **183** | 1 | 7 | 3 | 3 | 4.630 | 6.670 | 0.694 | 3.130 | 6.500 |
| **184** | 1 | 7 | 3 | 3 | 4.500 | 6.530 | 0.689 | 3.240 | 6.230 |
| **185** | 1 | 7 | 3 | 3 | 4.530 | 6.480 | 0.699 | 3.200 | 6.420 |
| **186** | 1 | 7 | 3 | 3 | 4.590 | 6.650 | 0.690 | 3.390 |  |
| **187** | 1 | 7 | 3 | 3 | 4.620 | 6.560 | 0.704 |  |  |
| **188** | 1 | 7 | 6 | 4 | 4.620 | 6.710 | 0.689 | 3.150 | 6.610 |
| **189** | 1 | 7 | 6 | 4 | 4.540 | 6.590 | 0.689 | 3.300 | 6.550 |
| **190** | 1 | 7 | 6 | 4 | 4.610 | 6.570 | 0.702 | 3.200 | 6.550 |
| **191** | 1 | 7 | 6 | 4 | 4.570 | 6.650 | 0.687 | 3.200 | 6.410 |
| **192** | 1 | 7 | 6 | 4 | 4.550 | 6.610 | 0.688 | 3.200 |  |
| **193** | 1 | 7 | 9 | 5 | 4.680 | 6.670 | 0.702 | 3.140 | 6.590 |
| **194** | 1 | 7 | 9 | 5 | 4.490 | 6.540 | 0.687 | 3.350 | 6.570 |
| **195** | 1 | 7 | 9 | 5 | 4.640 | 6.570 | 0.706 | 3.390 | 6.460 |
| **196** | 1 | 7 | 9 | 5 | 4.630 | 6.510 | 0.711 | 3.550 |  |
| **197** | 1 | 7 | 14 | 6 | 4.800 | 6.730 | 0.713 | 3.350 | 6.800 |
| **198** | 1 | 7 | 14 | 6 | 4.630 | 6.560 | 0.706 | 3.500 | 6.760 |
| **199** | 1 | 7 | 14 | 6 | 4.670 | 6.530 | 0.715 | 3.640 | 6.780 |
| **200** | 1 | 7 | 14 | 6 | 4.770 | 6.550 | 0.728 | 3.560 |  |
| **201** | 1 | 7 | 14 | 6 | 4.760 | 6.650 | 0.716 | 3.440 |  |
| **202** | 1 | 7 | 14 | 6 | 4.630 | 6.540 | 0.708 | 3.660 |  |
| **203** | 1 | 7 | 20 | 7 | 4.750 | 6.660 | 0.713 | 3.560 | 6.610 |
| **204** | 1 | 7 | 20 | 7 | 4.720 | 6.600 | 0.715 | 3.470 | 6.500 |
| **205** | 1 | 7 | 20 | 7 | 4.700 | 6.660 | 0.706 | 3.410 | 6.590 |
| **206** | 1 | 7 | 20 | 7 | 4.620 | 6.630 | 0.697 | 3.320 | 6.610 |
| **207** | 1 | 7 | after | 8 | 4.970 | 6.810 | 0.730 | 3.810 | 6.740 |
| **208** | 1 | 7 | after | 8 | 4.870 | 6.610 | 0.737 | 3.880 | 6.700 |
| **209** | 1 | 7 | after | 8 | 5.090 | 6.750 | 0.754 | 3.820 | 6.670 |
| **210** | 1 | 7 | after | 8 | 4.980 | 6.880 | 0.724 |  |  |
| **211** | 1 | 7 | after | 8 | 4.920 | 6.830 | 0.720 |  |  |
| **212** | 2 | 8 | before | 2 | 5.180 | 6.610 | 0.784 | 4.440 | 6.680 |
| **213** | 2 | 8 | before | 2 | 5.120 | 6.600 | 0.776 | 4.530 | 6.410 |
| **214** | 2 | 8 | before | 2 | 5.140 | 6.590 | 0.780 | 4.440 | 6.620 |
| **215** | 2 | 8 | before | 2 | 5.130 | 6.610 | 0.776 | 4.610 |  |
| **216** | 2 | 8 | 3 | 3 | 5.030 | 6.530 | 0.770 | 4.340 | 6.960 |
| **217** | 2 | 8 | 3 | 3 | 4.990 | 6.450 | 0.774 | 4.180 | 6.950 |
| **218** | 2 | 8 | 3 | 3 | 5.010 | 6.480 | 0.773 | 4.170 | 6.980 |
| **219** | 2 | 8 | 3 | 3 | 4.940 | 6.400 | 0.772 | 4.140 |  |
| **220** | 2 | 8 | 6 | 4 | 5.160 | 6.660 | 0.775 | 4.360 | 6.640 |
| **221** | 2 | 8 | 6 | 4 | 5.070 | 6.430 | 0.788 | 4.470 | 6.570 |
| **222** | 2 | 8 | 6 | 4 | 5.120 | 6.500 | 0.788 | 4.480 | 6.570 |
| **223** | 2 | 8 | 6 | 4 | 5.460 | 6.550 | 0.834 | 4.540 |  |
| **224** | 2 | 8 | 9 | 5 | 5.080 | 6.640 | 0.765 | 4.000 | 6.680 |
| **225** | 2 | 8 | 9 | 5 | 5.020 | 6.520 | 0.770 | 4.340 | 6.690 |
| **226** | 2 | 8 | 9 | 5 | 5.050 | 6.530 | 0.773 | 4.150 | 6.630 |
| **227** | 2 | 8 | 14 | 6 | 5.180 | 6.740 | 0.769 | 4.320 | 6.940 |
| **228** | 2 | 8 | 14 | 6 | 5.030 | 6.460 | 0.779 | 4.210 | 6.820 |
| **229** | 2 | 8 | 14 | 6 | 5.120 | 6.690 | 0.765 | 4.400 | 6.850 |
| **230** | 2 | 8 | 14 | 6 | 5.140 | 6.650 | 0.773 | 4.460 |  |
| **231** | 2 | 8 | 14 | 6 | 5.140 | 6.620 | 0.776 |  |  |
| **232** | 2 | 8 | 20 | 7 | 5.270 | 6.730 | 0.783 | 4.580 | 6.980 |
| **233** | 2 | 8 | 20 | 7 | 5.210 | 6.630 | 0.786 | 4.700 | 6.890 |
| **234** | 2 | 8 | 20 | 7 | 5.250 | 6.660 | 0.788 | 4.590 | 6.980 |
| **235** | 2 | 8 | 20 | 7 | 5.250 | 6.720 | 0.781 | 4.690 |  |
| **236** | 2 | 8 | after | 8 | 5.450 | 6.790 | 0.803 | 4.860 | 6.990 |
| **237** | 2 | 8 | after | 8 | 5.290 | 6.650 | 0.795 | 5.140 | 6.850 |
| **238** | 2 | 8 | after | 8 | 5.280 | 6.780 | 0.779 | 4.620 | 6.990 |
| **239** | 2 | 8 | after | 8 | 5.310 | 6.700 | 0.793 | 4.840 |  |
| **240** | 2 | 9 | before | 2 | 4.900 | 5.530 | 0.886 | 5.950 | 5.420 |
| **241** | 2 | 9 | before | 2 | 4.770 | 5.400 | 0.883 | 5.960 | 5.280 |
| **242** | 2 | 9 | before | 2 | 4.850 | 5.440 | 0.892 | 6.060 | 5.390 |
| **243** | 2 | 9 | before | 2 | 4.820 | 5.430 | 0.888 | 5.940 |  |
| **244** | 2 | 9 | 3 | 3 | 4.950 | 5.550 | 0.892 | 5.770 | 5.370 |
| **245** | 2 | 9 | 3 | 3 | 4.760 | 5.440 | 0.875 | 5.980 | 5.360 |
| **246** | 2 | 9 | 3 | 3 | 4.820 | 5.520 | 0.873 | 6.100 | 5.270 |
| **247** | 2 | 9 | 3 | 3 | 4.850 | 5.410 | 0.896 | 5.520 | 5.230 |
| **248** | 2 | 9 | 6 | 4 | 5.100 | 5.670 | 0.899 | 6.370 | 5.670 |
| **249** | 2 | 9 | 6 | 4 | 5.080 | 5.640 | 0.901 | 6.440 | 5.510 |
| **250** | 2 | 9 | 6 | 4 | 5.030 | 5.600 | 0.898 | 6.460 | 5.660 |
| **251** | 2 | 9 | 6 | 4 | 5.030 | 5.590 | 0.900 | 6.360 |  |
| **252** | 2 | 9 | 9 | 5 | 5.150 | 5.730 | 0.899 | 6.540 | 5.610 |
| **253** | 2 | 9 | 9 | 5 | 5.040 | 5.690 | 0.886 | 6.180 | 5.600 |
| **254** | 2 | 9 | 9 | 5 | 5.060 | 5.600 | 0.904 | 6.330 | 5.620 |
| **255** | 2 | 9 | 14 | 6 | 5.250 | 5.830 | 0.901 | 6.480 | 5.800 |
| **256** | 2 | 9 | 14 | 6 | 5.120 | 5.700 | 0.898 | 6.470 | 5.710 |
| **257** | 2 | 9 | 14 | 6 | 5.190 | 5.820 | 0.892 | 6.410 | 5.710 |
| **258** | 2 | 9 | 14 | 6 | 5.160 | 5.770 | 0.894 | 6.570 |  |
| **259** | 2 | 9 | 20 | 7 | 5.020 | 5.640 | 0.890 | 6.030 | 5.690 |
| **260** | 2 | 9 | 20 | 7 | 4.830 | 5.440 | 0.888 | 6.160 | 5.580 |
| **261** | 2 | 9 | 20 | 7 | 4.930 | 5.540 | 0.890 | 6.110 | 5.640 |
| **262** | 2 | 9 | 20 | 7 | 4.950 | 5.520 | 0.897 | 6.250 | 5.640 |
| **263** | 2 | 9 | after | 8 | 5.160 | 5.860 | 0.881 | 6.580 | 5.630 |
| **264** | 2 | 9 | after | 8 | 5.160 | 5.780 | 0.893 | 6.250 | 5.560 |
| **265** | 2 | 9 | after | 8 | 5.110 | 5.780 | 0.884 | 6.320 | 5.590 |
| **266** | 2 | 9 | after | 8 | 5.040 | 5.660 | 0.890 | 6.280 |  |
| **267** | 2 | 10 | before | 2 | 4.140 | 5.040 | 0.821 | 3.380 | 4.740 |
| **268** | 2 | 10 | before | 2 | 3.920 | 4.940 | 0.794 | 3.710 | 4.730 |
| **269** | 2 | 10 | before | 2 | 4.020 | 4.850 | 0.829 | 3.890 | 4.670 |
| **270** | 2 | 10 | before | 2 | 4.090 | 4.880 | 0.838 | 3.800 |  |
| **271** | 2 | 10 | 3 | 3 | 3.670 | 4.710 | 0.779 | 2.980 | 4.800 |
| **272** | 2 | 10 | 3 | 3 | 3.620 | 4.700 | 0.770 | 3.140 | 4.680 |
| **273** | 2 | 10 | 3 | 3 | 3.630 | 4.560 | 0.796 | 2.820 | 4.730 |
| **274** | 2 | 10 | 3 | 3 | 3.550 | 4.700 | 0.755 | 3.100 |  |
| **275** | 2 | 10 | 6 | 4 | 3.940 | 4.860 | 0.811 | 3.620 | 4.780 |
| **276** | 2 | 10 | 6 | 4 | 3.870 | 4.850 | 0.798 | 3.450 | 4.740 |
| **277** | 2 | 10 | 6 | 4 | 3.900 | 4.810 | 0.811 | 3.460 | 4.740 |
| **278** | 2 | 10 | 6 | 4 | 3.810 | 4.850 | 0.786 | 3.250 |  |
| **279** | 2 | 10 | 9 | 5 | 3.810 | 4.770 | 0.799 | 3.200 | 4.570 |
| **280** | 2 | 10 | 9 | 5 | 3.670 | 4.640 | 0.791 | 3.360 | 4.570 |
| **281** | 2 | 10 | 9 | 5 | 3.750 | 4.630 | 0.810 | 3.360 | 4.600 |
| **282** | 2 | 10 | 14 | 6 | 3.930 | 4.680 | 0.840 | 3.510 | 4.760 |
| **283** | 2 | 10 | 14 | 6 | 3.800 | 4.670 | 0.814 | 3.910 | 4.720 |
| **284** | 2 | 10 | 14 | 6 | 3.790 | 4.430 | 0.856 | 3.650 | 4.730 |
| **285** | 2 | 10 | 14 | 6 | 3.750 | 4.480 | 0.837 | 3.670 | 4.630 |
| **286** | 2 | 10 | 14 | 6 | 3.810 | 4.630 | 0.823 | 3.870 |  |
| **287** | 2 | 10 | 14 | 6 | 3.830 | 4.570 | 0.838 | 3.630 |  |
| **288** | 2 | 10 | 20 | 7 | 3.940 | 4.710 | 0.837 | 3.660 | 4.680 |
| **289** | 2 | 10 | 20 | 7 | 3.850 | 4.640 | 0.830 | 3.860 | 4.620 |
| **290** | 2 | 10 | 20 | 7 | 3.910 | 4.640 | 0.843 | 3.850 | 4.660 |
| **291** | 2 | 10 | 20 | 7 | 3.890 | 4.670 | 0.833 | 3.730 | 4.610 |
| **292** | 2 | 10 | after | 8 | 4.170 | 4.950 | 0.842 | 4.220 | 4.650 |
| **293** | 2 | 10 | after | 8 | 4.150 | 4.830 | 0.859 | 4.270 | 4.600 |
| **294** | 2 | 10 | after | 8 | 4.150 | 4.860 | 0.854 | 4.100 | 4.580 |
| **295** | 2 | 10 | after | 8 | 4.130 | 4.820 | 0.857 | 4.170 |  |
| **296** | 1 | 11 | before | 2 | 5.070 | 5.480 | 0.925 | 6.260 | 5.550 |
| **297** | 1 | 11 | before | 2 | 4.990 | 5.390 | 0.926 | 6.400 | 5.340 |
| **298** | 1 | 11 | before | 2 | 4.930 | 5.440 | 0.906 | 6.140 | 5.460 |
| **299** | 1 | 11 | before | 2 | 4.960 | 5.410 | 0.917 | 5.980 |  |
| **300** | 1 | 11 | 3 | 3 | 4.980 | 5.540 | 0.899 | 5.810 | 5.300 |
| **301** | 1 | 11 | 3 | 3 | 4.950 | 5.520 | 0.897 | 5.730 | 5.120 |
| **302** | 1 | 11 | 3 | 3 | 4.960 | 5.500 | 0.902 | 5.690 | 5.210 |
| **303** | 1 | 11 | 3 | 3 | 4.890 | 5.370 | 0.911 | 5.700 |  |
| **304** | 1 | 11 | 6 | 4 | 5.030 | 5.710 | 0.881 | 5.690 | 5.500 |
| **305** | 1 | 11 | 6 | 4 | 4.990 | 5.540 | 0.901 | 5.660 | 5.400 |
| **306** | 1 | 11 | 6 | 4 | 4.960 | 5.550 | 0.894 | 5.550 | 5.490 |
| **307** | 1 | 11 | 6 | 4 | 4.990 | 5.580 | 0.894 | 5.670 | 5.500 |
| **308** | 1 | 11 | 9 | 5 | 5.080 | 5.820 | 0.873 | 5.720 | 5.750 |
| **309** | 1 | 11 | 9 | 5 | 5.000 | 5.660 | 0.883 | 5.560 | 5.640 |
| **310** | 1 | 11 | 9 | 5 | 5.060 | 5.790 | 0.874 | 5.610 | 5.740 |
| **311** | 1 | 11 | 9 | 5 | 4.940 | 5.710 | 0.865 | 5.430 | 5.630 |
| **312** | 1 | 11 | 14 | 6 | 5.110 | 5.800 | 0.881 | 5.550 | 5.730 |
| **313** | 1 | 11 | 14 | 6 | 5.080 | 5.780 | 0.879 | 5.680 | 5.730 |
| **314** | 1 | 11 | 14 | 6 | 5.080 | 5.750 | 0.883 | 5.610 | 5.700 |
| **315** | 1 | 11 | 14 | 6 | 5.040 | 5.770 | 0.873 | 5.620 | 5.710 |
| **316** | 1 | 11 | 20 | 7 | 5.020 | 5.730 | 0.876 | 5.390 | 5.570 |
| **317** | 1 | 11 | 20 | 7 | 4.870 | 5.540 | 0.879 | 5.420 | 5.690 |
| **318** | 1 | 11 | 20 | 7 | 5.000 | 5.670 | 0.882 | 5.500 | 5.560 |
| **319** | 1 | 11 | 20 | 7 | 4.880 | 5.610 | 0.870 | 5.300 |  |
| **320** | 1 | 11 | 20 | 7 | 4.860 | 5.500 | 0.884 | 5.260 |  |
| **321** | 1 | 11 | 20 | 7 | 4.850 | 5.700 | 0.851 | 5.100 |  |
| **322** | 1 | 11 | after | 8 | 5.100 | 5.680 | 0.898 | 5.860 | 5.680 |
| **323** | 1 | 11 | after | 8 | 5.000 | 5.620 | 0.890 | 5.940 | 5.610 |
| **324** | 1 | 11 | after | 8 | 5.060 | 5.670 | 0.892 | 5.890 | 5.620 |
| **325** | 1 | 11 | after | 8 | 5.100 | 5.630 | 0.906 | 5.860 |  |
